# Multiplatform modeling of atrial fibrillation identifies phospholamban as a central regulator of cardiac rhythm

**DOI:** 10.1242/dmm.049962

**Published:** 2023-07-17

**Authors:** Anaïs Kervadec, James Kezos, Haibo Ni, Michael Yu, James Marchant, Sean Spiering, Suraj Kannan, Chulan Kwon, Peter Andersen, Rolf Bodmer, Eleonora Grandi, Karen Ocorr, Alexandre R. Colas

**Affiliations:** ^1^ Sanford Burnham Prebys Medical Discovery Institute, La Jolla, CA 92037, USA; ^2^ Department of Pharmacology, UC Davis, Davis, CA 95616, USA; ^3^ Johns Hopkins University, Baltimore, MD 21205, USA

**Keywords:** Cardiac disease modeling, Atrial fibrillation, Human iPSC-derived atrial-like cardiomyocytes, Computational modeling, High-throughput electrophysiology, Single-cell resolution, *Drosophila*

## Abstract

Atrial fibrillation (AF) is a common and genetically inheritable form of cardiac arrhythmia; however, it is currently not known how these genetic predispositions contribute to the initiation and/or maintenance of AF-associated phenotypes. One major barrier to progress is the lack of experimental systems to investigate the effects of gene function on rhythm parameters in models with human atrial and whole-organ relevance. Here, we assembled a multi-model platform enabling high-throughput characterization of the effects of gene function on action potential duration and rhythm parameters using human induced pluripotent stem cell-derived atrial-like cardiomyocytes and a *Drosophila* heart model, and validation of the findings using computational models of human adult atrial myocytes and tissue. As proof of concept, we screened 20 AF-associated genes and identified phospholamban loss of function as a top conserved hit that shortens action potential duration and increases the incidence of arrhythmia phenotypes upon stress. Mechanistically, our study reveals that phospholamban regulates rhythm homeostasis by functionally interacting with L-type Ca^2+^ channels and NCX. In summary, our study illustrates how a multi-model system approach paves the way for the discovery and molecular delineation of gene regulatory networks controlling atrial rhythm with application to AF.

## Teaser

**Summary:** We assembled and validated a novel multi-model system platform that enables the rapid evaluation of the pro-arrhythmogenic potential of genes associated with human arrhythmias such as atrial fibrillation.

## INTRODUCTION

Atrial fibrillation (AF) is the most common form of sustained cardiac arrhythmia in humans (Du et al., 2017). At the whole-heart level, a central feature of AF is very rapid and uncoordinated atrial activity; at the cellular level, the mechanism maintaining arrhythmia often arises from a ‘vulnerable substrate’, which consists of action potential duration (APD) prolongation or shortening events (Christophersen et al., 2013). Such vulnerable substrates are thought to be caused by genetic predispositions, cardiac remodeling caused by heart disease, aging and/or altered regulation by neurohormonal factors (Campuzano and Brugada, 2009; Chen et al., 2014; Lip et al., 2016; Weng et al., 2017). In this context, linkage analysis in familial cases of AF (Chen et al., 2003; Hodgson-Zingman et al., 2008; Olson et al., 2006) as well as genome-wide associated studies (GWAS) in the general population (Christophersen et al., 2017; Nielsen et al., 2018b; Roselli et al., 2018) have elucidated some of the genetic underpinnings associated with the disease. As a result, close to 140 genetic loci linked to >200 genes have been identified (Fatkin et al., 2017; Roselli et al., 2018, 2020); however, none of these genes have been validated as disease causing in the general population, limiting drug discovery efforts. In this context, a major barrier to progress is the lack of experimental platforms/strategies to enable rapid establishment of the causal links between gene function and AF-associated phenotypes (electrical remodeling, arrhythmia).

Among the variety of models available to evaluate AF-associated genes, the four-chambered mouse heart has been extensively used to establish functional links between genes or genetic loci and rhythm phenotypes (Lozano-Velasco et al., 2016; Nadadur et al., 2016; Temple et al., 2005; van Ouwerkerk et al., 2019; Wang et al., 2010; Zhang et al., 2019). However, despite high proteome homology with humans and ability to manipulate the genome, the substantial electrophysiological differences [fast resting rate, short AP duration and triangular shape, species-specific K^+^ channels (Kaese and Verheule, 2012)], relatively long lifespan (years) and low-throughput capacity of methods to retrieve electrophysiological parameters limit the use of mice as a primary model for gene discovery related to AF.

In contrast to mice, flies have a short generation time (∼10 days), and established automated kinetic imaging techniques (Fink et al., 2009; Klassen et al., 2017), coupled with available functional genomic resources (e.g. flybase.org; Mohr et al., 2014), enable the rapid evaluation of the effects of gene function on rhythm parameters at the whole-heart level. In addition, although the fly heart structurally differs from that of vertebrates, the fundamental mechanisms of development and function are remarkably conserved, including a common transcriptional regulatory network (Bodmer, 1995; Cripps and Olson, 2002), shared protein composition (Cammarato et al., 2011), and electrical and metabolic properties (Diop and Bodmer, 2015; Ocorr et al., 2007c, 2014). Thus, the adult fly heart represents an appealing model to rapidly evaluate the role of evolutionarily conserved genes for their ability to regulate cardiac rhythm, although a limitation to this model is the lack of atrial specificity.

The advent of induced pluripotent stem cell (iPSC) technology (Takahashi et al., 2007; Takahashi and Yamanaka, 2006) and protocols enabling the generation of subtype-specific cardiomyocytes (CMs) (Burridge et al., 2014; Cunningham et al., 2017; Devalla et al., 2015; Yu et al., 2018) provide a unique experimental access to human atrial myocyte biology. In addition, the recent development of high-throughput (HT) kinetic imaging techniques (Cerignoli et al., 2012; McKeithan et al., 2017), fluorescent Ca^2+^ and voltage-sensing indicators (Liu and Miller, 2020; Paredes et al., 2008), coupled with available functional genomic resources [small interfering RNAs (siRNAs), CRISPR/Cas9 guide libraries], enable large-scale exploration of the effects of gene function on human cardiac electrophysiological (voltage and Ca^2+^ transients kinetics) and rhythm (rhythmicity, beat rate) parameters (Elmen et al., 2020; Murphy et al., 2021). Although human iPSC (hiPSC)-derived atrial-like cardiomyocytes (ACMs) are well suited to identify atrial-specific and cell-autonomous rhythm-regulating mechanisms (Devalla et al., 2016; Marczenke et al., 2017a,b), the relative immaturity of hiPSC-derived CMs (Cho et al., 2017; Yang et al., 2014) and inherent lack of tissue level integration might limit translation of the findings to the adult human heart. In sum, single-model approaches are limited in their ability to validate large cohorts of AF-associated genes, indicating the necessity to develop alternative strategies to improve AF gene validation.

Based on these observations, we reasoned that combining assays with human, atrial and whole-organ relevance that also have HT functional genomics capacity could enhance our ability to rapidly establish causal links between AF-associated genes and arrhythmia phenotypes. To establish such a platform, we developed a human-relevant assay that measures APD in ACMs with single-cell resolution. In parallel, we optimized a fly cardiac function assay that measures contraction duration [systolic interval (SI)], as a surrogate measurement for APD. As proof of concept, we screened a cohort of 20 AF-associated genes and identified phospholamban (*PLN*) loss of function as a top conserved hit that significantly shortens APD in ACMs, human atrial myocytes (HAMs) and fly CMs. Remarkably, although *PLN* knockdown (KD) was not sufficient to induce arrhythmia phenotypes on its own, addition of environmental stressors (i.e. fibroblasts, β-adrenergic stimulation) increased the generation of irregular beat-to-beat intervals and delayed afterdepolarizations (DADs), compared to those in controls, and triggered action potentials (APs). Finally, to delineate the mechanism underlying *PLN* KD-dependent arrhythmia, we used a logistic regression approach in HAM populations, and predicted that PLN functionally interacts with both NCX (loss of function) and L-type Ca^2+^ channels (LTCCs; gain of function) to mediate these arrhythmic phenotypes. Consistent with our predictions, co-KD of *PLN* and *NCX* in ACMs and flies led to increased arrhythmic events, whereas treatment of ACMs with the LTCC inhibitor verapamil reverted these phenotypes.

## RESULTS

### Integrated multi-model system platform to identify rhythm-regulating genes

To phenotypically assess AF-associated genes, we established a novel phenotypic platform, enabling us to study the effects of gene function on APD and rhythm parameters in HT in ACMs and flies.

#### Single-cell and HT assessment of APD and rhythm parameters in ACMs

**Fig. 1.**
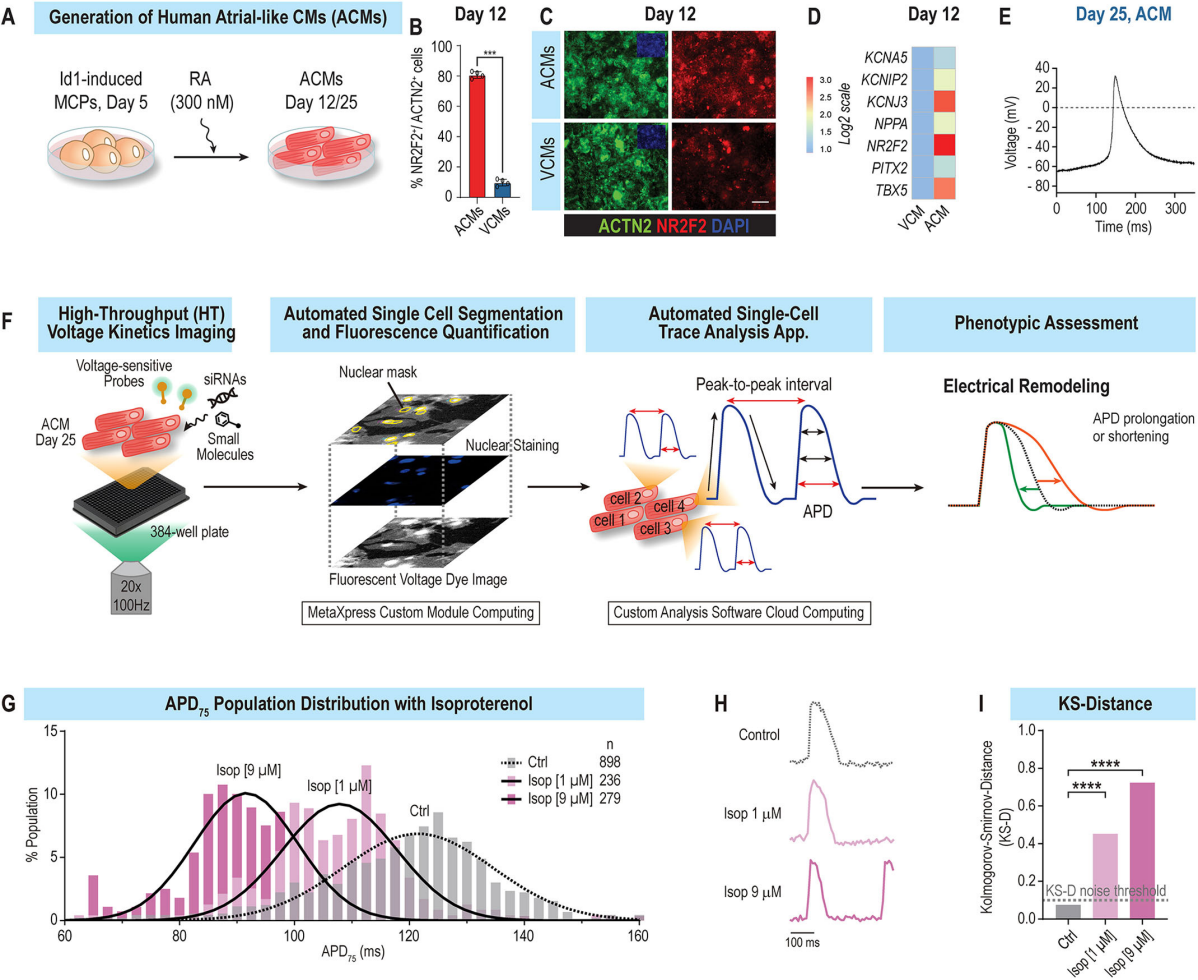
**Atrial-like cardiomyocyte (ACM) platform.** (A) Schematic representation of the ACM differentiation protocol. To promote atrial differentiation, day 5 cardiac progenitors were treated with 300 nM retinoic acid (RA) and subsequently cultured until day 12 or 25. MCP, multipotent cardiac progenitor. (B) RA treatment efficiently induces the generation of atrial-like NR2F2^+^ beating cardiomyocytes (CMs) (∼80% of NR2F2^+^, ACTN2^+^). VCM, ventricular CM. (C) Representative immunofluorescence images showing overexpression of NR2F2^+^ (red) and ACTN2^+^ (green) cells in ACMs. Scale bar: 100 μm. (D) Heatmap of atrial genes enriched in day 12 ACMs compared to VCMs. (E) Patch-clamp experiments show that ACMs generate atrial-like triangular-shaped action potentials (APs). (F) Schematic representation of single-cell and high-throughput (HT) platform to measure AP duration (APD) parameters in ACMs. (G) Population distribution of APD_75_ values from ACMs treated with increasing doses of isoproterenol (Isop), showing dose-dependent APD shortening. (H) Single AP traces of median APD_75_ for each condition. (I) Kolmogorov–Smirnov distance (KS-D) values for control (Ctrl) and Isop-treated ACMs. ***P<0.001, ****P<0.0001.

To study the molecular basis of chamber-specific electrical disorders such as AF, Id1-overexpressing cardiac progenitors (CPs) were used to generate ACMs as described previously (Cunningham et al., 2017; Yu et al., 2018). Treatment of Id1-induced CPs with a single dose of retinoic acid (300 nM) efficiently promoted the generation of atrial-like, NR2F2^+^ beating CMs (∼80% were NR2F2^+^, ACTN2^+^) (Fig. 1A-C). Consistent with an atrial-like identity, induced ACMs also expressed atria-enriched (Devalla et al., 2015; Uhlen et al., 2015) transcription factors (NR2F2, TBX5, ZNF385B), ion channel genes *KCNA5* (encoding Kv1.5) and *KCNJ3* (encoding Kir 3.1), ligands (NPPA, NPPB) and receptors (EGR1/2, PDGFRA), at day 12 and day 25 of differentiation (Fig. 1D; Fig. S1A,B). Electrophysiologically, ACMs typically generated short (∼120 ms) and triangular APs (Fig. 1E), whereas untreated Id1-induced CPs generated CMs that displayed longer AP (∼200 ms) with a plateaued phase 2 (Fig. S1C), reminiscent of a ventricular-like identity (Ng et al., 2010). Moreover, and consistent with an atrial cell fate, ACMs also displayed shorter Ca^2+^ transient durations [CTDs; CTD_50_ (50% duration of the Ca^2+^ transient) and CTD_75_] compared to those of ventricular CMs (VCMs) (Fig. S1D-G) (Ng et al., 2010).

Next, to facilitate the characterization of AF-associated arrhythmia phenotypes in ACMs, we developed an imaging platform that automatically tracks and quantifies AP and rhythm parameters in HT with single-cell resolution (Fig. 1F). To retrieve AP and rhythm parameters, ACMs were co-labeled with a voltage dye (VF2.1.Cl) and a nuclear dye (Hoechst 33258) as described in McKeithan et al. (2017). For each condition, one image of the Hoechst dye was collected, followed by a 5-s acquisition of the voltage dye channel at 100 Hz. Next, using a custom algorithm developed on ImageXpress, each cell in the field of view was segmented using the Hoechst dye topological information and each cell mask was propagated to the ‘voltage dye channel’, thereby enabling the quantification of voltage-dependent fluorescence variation over time with single-cell resolution. To retrieve the electrophysiological parameters, we developed a cloud-based trace analysis application that automatically processes each AP trace and retrieves median and standard deviation values [APD_10_ (10% duration of the AP), APD_25_, APD_50_, APD_75_, APD_90_; T_25-75_ (duration between 25% and 75% of the Ca^2+^ transient), T_75-25_; Vmax up (maximum upstroke velocity of the action potential) and down (maximum downstroke velocity of the action potential); beat rate; peak-to-peak interval; and rhythm regularity index] for each cell. This platform enabled us to automatically record, quantify and analyze AP and rhythm parameters in less than 2 min per condition.

To test the platform's ability to identify APD modulators, we infused ACMs with isoproterenol, a non-selective β-adrenergic agonist, known to shorten APD and increase beat rate in hiPSC-derived CMs (McKeithan et al., 2017). Consistent with previous studies, increasing doses of isoproterenol caused a dose-dependent shortening of median APD_75_ values, from 121.3 ms (untreated) to 108.6 ms (1 µM) and 91.2 ms (9 µM) (Fig. 1G,H). To measure whether isoproterenol treatment had a significant effect on APD at the whole-cell-population level, we used the Kolmogorov–Smirnov distance test (KS-D) (Dal Molin et al., 2017; Delmans and Hemberg, 2016; Feng et al., 2009; Gaber et al., 2013), which enables the quantification and comparison of the distributional differences of binary features such as APD_75_ or beat rate. Consistent with median APD_75_ values, increasing doses of isoproterenol (1 and 9 µM) led to an increase in APD_75_ KS-D value to ∼0.4 and 0.7, respectively, compared to control (Fig. 1I). Similarly, to quantify cellular manifestations of arrhythmia, we defined an arrhythmia index (AI) that quantifies beat-to-beat interval irregularities as a metric for arrhythmically beating cells (Fig. S2A). In this context, we determined that AP trains with AI values <20 generally describe regular beating patterns, whereas AP trains with AI values >20 mark generally mark irregular beating patterns (Fig. S2B). Finally, to benchmark our platform for arrythmia-associated phenotypes, we tested the role of dofetilide, a class III anti-arrhythmic, which selectively blocks the rapid component of the delayed rectifier outward K^+^. At therapeutic dose, dofetilide prolongs the APD and subsequently increases the refractory period, thereby mediating its anti-arrhythmic effect (Geng et al., 2020). Conversely, at higher doses, dofetilide increases the incidence of arrhythmia phenotypes such as early afterdepolarizations (EADs) (Jaiswal and Goldbarg, 2014; McKeithan et al., 2017). Consistent with these observations, ACMs treated with a low dose of dofetilide (33 nM) displayed a reduced AI compared to that of control, whereas those treated with a high dose of dofetilide (100 nM) exhibited a markedly increased percentage of cells with AI values >20 (from 4% to 67%) and associated KS-D value of 0.7 (Fig. S2C-F). Collectively, these data show that this new phenotypic platform enables the HT and automated quantification of APD and rhythm parameters in ACMs with single-cell resolution.

#### HT measurement of APD and rhythm in a *Drosophila* cardiac platform

**Fig. 2.**
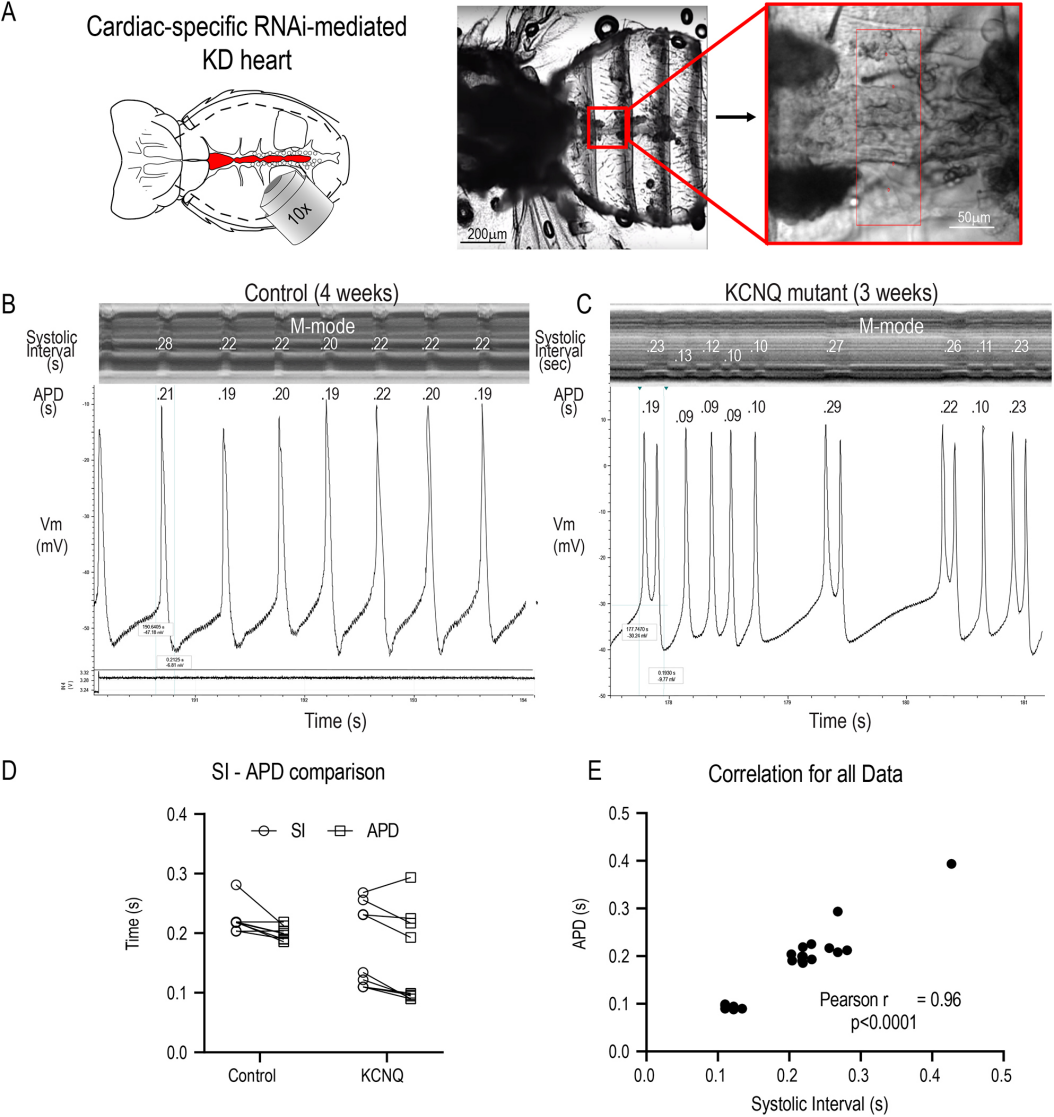
**Fly heart platform.** (A) Schematic of the fly thorax and abdomen (left; heart tube is shown in red) and image of semi-intact preparation (middle) with a single cardiac chamber (red box), shown at higher magnification on the right. KD, knockdown; RNAi, RNA interference. (B,C) Simultaneous optical and electrophysiological recordings from beating hearts. M-modes from optical recordings are shown at the top with the corresponding AP traces below. APDs and systolic intervals (SIs) are shown in seconds. The lower window in B shows the voltage trace generated by the image capture software that was used to synchronize the optical and electrical recordings. (D) SIs are paired with their corresponding APs. (E) The Pearson correlation coefficient for the combined data in D showed a significant correlation between SIs and APDs (*r*=0.96, *P*<0.0001).

To assess AF-associated mechanisms at the whole-organ level using a *Drosophila* model, we used high-speed video recording of heart movements in *in situ* preparations (Fig. 2A). Heart function was quantified as previously described (Fink et al., 2009; Vogler and Ocorr, 2009), providing precise measurements of heart period (R-R interval), SI, arrhythmicity and fractional shortening/contractility in a functioning heart. We have previously shown that most of the key cardiac ion channels present in human hearts are also present and functional in the fly heart (Ocorr et al., 2007c, 2017; Table S1). Importantly, our previous studies using simultaneous optical and intracellular recordings demonstrated a direct 1:1 correlation between myocardial cell depolarization and heart wall movement. It is important to note that the fly heart is composed of a single layer of myocardial cells and any heart wall movement is an immediate reflection of the contractile state of component myocardial cells. Thus, we quantified APD and the corresponding SI from simultaneous electrical and optical recordings, respectively, from hearts of middle-aged wild-type controls and KCNQ mutants and found a strong correlation (*r*=0.96, *P*<0.0001) between APD and SI (Fig. 2B-E). Therefore, we used cardiac contraction/relaxation movements as surrogates for APD (Cammarato et al., 2015). In this context, cardiac-specific gene KD was achieved using a Gal4-based system (Brand and Perrimon, 1993) that drives expression of double-stranded RNA interference (dsRNAi) in a cardiac-specific manner. AF is an aging-related disease, and the fly provides an opportunity to examine the effects of cardiac gene KD at young, middle and old ages (∼1, 3 and 5 weeks, respectively). Finally, ∼75% of human disease-causing genes are represented in the fly, usually as single copies, and often cause remarkably similar disease phenotypes (Bier, 2005; Bier and Bodmer, 2004). For example, flies with mutations in the *KNCQ* gene exhibited a torsades de pointes-like phenotype, and age-dependent increases in arrhythmia in wild-type flies was associated with reduced expression of genes encoding KATP and KCNQ channels (Ocorr et al., 2007a). We also showed that electrical remodeling increased with age and was exacerbated by KCNQ and hERG (also known as KCNH2) mutations (Ocorr et al., 2017).

### Functional screen of AF-associated genes identifies *PLN* loss of function as a major driver of APD and contraction interval shortening

**Fig. 3.**
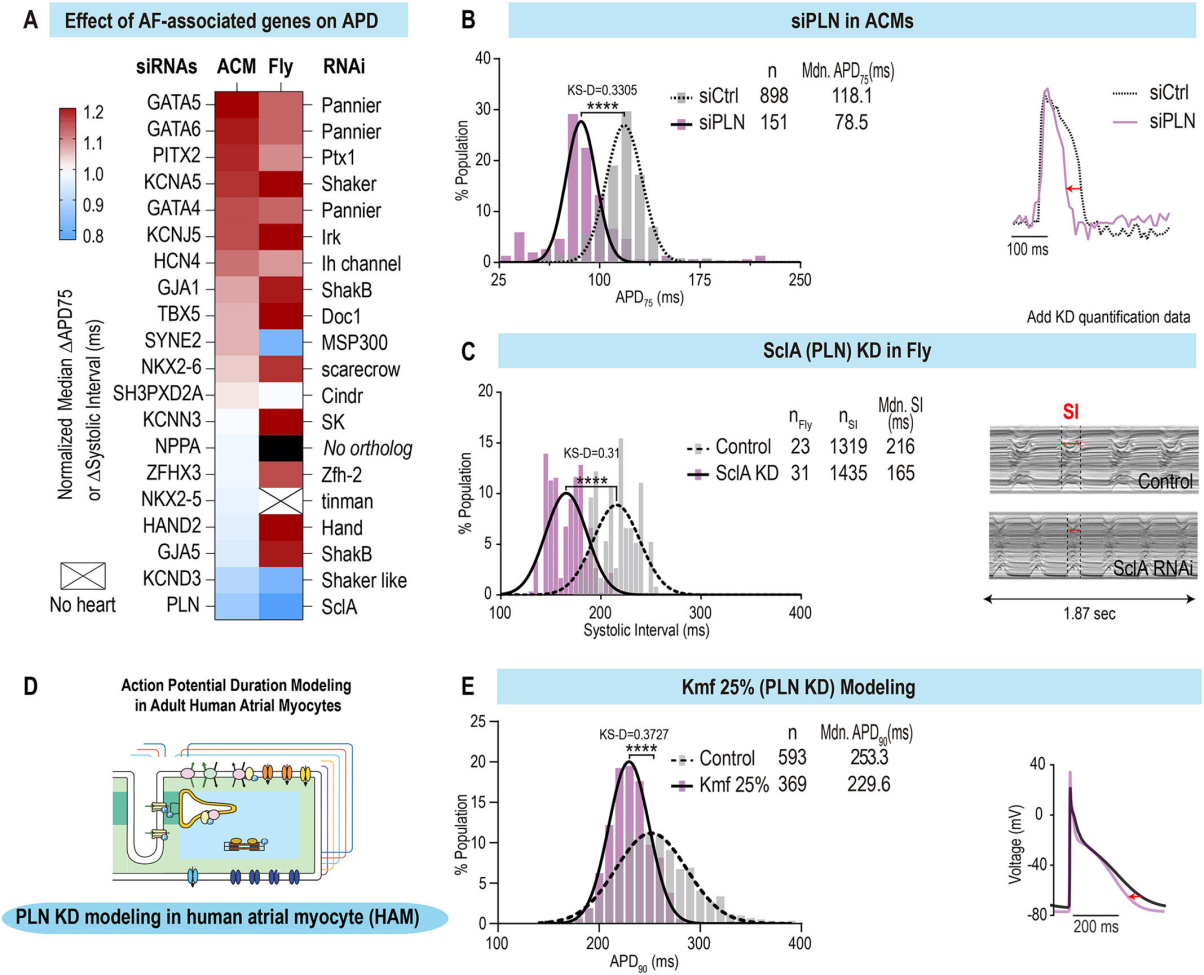
**Loss-of-function screen of atrial fibrillation (AF)-associated genes identifies conserved modulators of APD and SI in a multi-model system platform.** (A) Heatmap showing the normalized effects of AF-associated gene loss of function on APD and SI in ACMs and flies. (B) Population distribution of APD_75_ values for siControl and siPLN-transfected ACMs (left) and representative AP traces (right) showing the shortening effect of siPLN. Mdn, median. (C) Population distribution of SIs in control versus *SclA* KD conditions in flies (left) and representative M-modes (right), showing the SI shortening effect for *SclA* KD. (D) Schematic representation of APD modeling in human atrial monocytes (HAMs). (E) Population distribution of APD_90_ values for control and Michaelis constant (Km) of SERCA to cytosolic Ca^2+^ (forward pumping) (Kmf) 25% (*PLN* KD) in HAMs (left) and representative AP traces (right), showing the shortening effect on APD of simulated *PLN* KD. ****P<0.0001 (KS-D).

To evaluate the ability of the platform to identify AF-associated genes and mechanisms, we first assessed the expression of genes previously associated with AF (Fatkin et al., 2017) by RNA sequencing (RNA-seq) of day 12 and day 25 ACMs. The results revealed that most AF candidate genes were expressed in ACMs at moderate-to-high levels [from 0.1 to >100 reads per kilobase of transcript, per million mapped reads (RPKM)] (Fig. S3A) and most are also expressed in the fly heart (Table S1). Next, we selected 20 genes that had been identified in rare variant familial AF studies and/or as having single-nucleotide polymorphisms (SNPs) reported in GWAS (Fig. S3B; Fatkin et al., 2017). To evaluate their effect on APD, we transfected siRNAs directed against these 20 AF-associated genes into day 25 ACMs and measured voltage variation over time with single-cell resolution. Remarkably, APD_75_ population measurements revealed that nine of 20 siRNAs induced electrical remodeling (KS-D value >0.25, *P*<0.001) (Fig. 3A; Fig. S3C, Tables S2 and S3). Interestingly, the screen identified two phenotypes: prolongation or shortening of APD. Among these, downregulation of *GATA5*, *GATA6*, *PITX2* and *KCNA5* significantly prolonged APD, whereas KD of *PLN* and *KCND3* shortened APD.

In parallel, we screened 24 fly genes, orthologous to 17 of the 20 AF-associated genes. Genes were knocked down using a heart-specific driver [Hand4.2-Gal4 (Sellin et al., 2006)] crossed to UAS-candidate gene RNA interference (RNAi) lines. Progeny of the crosses were aged to 3-week-old (middle-aged) flies, and heart function was characterized. Thirteen of the genes tested exhibited significantly altered SIs and/or rhythm phenotypes in the fly cardiac model, and seven of these overlapped with the genes affecting APD in the ACMs (*P*<0.001; Fig. 3A). In particular, cardiac-specific KD of *KNCJ5*/*Irk3*, *GATA4-6*/*pnr*, *PITX2*/*Ptx1* and *KCNA5*/*Sh* resulted in prolonged SIs, consistent with the increased APD observed for ACMs. Cardiac KD of *KCND3*/*Shal* and *PLN*/*SclA* significantly shortened SIs, paralleling the reductions in APD observed in ACMs. Although no significant changes in arrhythmicity were observed in the ACMs, we did observe increased arrhythmicity [AI and median absolute deviation (MAD) parameters] in flies in response to cardiac KD of three genes (*Irk2*, *pnr* and *SK*; Wilcoxcon-ranked sum test, *P*<0.001) in *Drosophila*.

Although there is evidence that APD prolongation is associated with AF (Nielsen et al., 2013; Olson et al., 2006), APD shortening is thought to be the most common mechanism underlying the onset and maintenance of AF (Teh et al., 2012; Wakili et al., 2011). We therefore focused on the gene KD that induced the strongest APD shortening phenotype. Remarkably, in both ACM and fly heart platforms, reduced *PLN*/*SclA* function consistently led to the strongest APD and SI shortening phenotype. In ACMs, *PLN* KD caused a significant shortening of median APD_75_ values, from 118.1 ms to 78.5 ms (∼−40 ms) (KS-D value=0.3305, *P*<0.0001) and with CTD (Fig. 3B; Fig. S3D). Similarly, cardiac-specific KD of *SclA* (the *PLN* homolog in fly) significantly shortened contractions of the fly heart from 216 ms to 165 ms (Fig. 3C).

To validate the phenotypic platform findings, we next employed a computational modeling approach of adult HAMs (Fig. 3D) (Grandi et al., 2011). Briefly, we generated a population of 600 HAMs with randomly varying model parameters to mimic natural cell-to-cell heterogeneity observed in cardiac tissues (Morotti et al., 2017; Ni et al., 2018) and simulated *PLN* KD by increasing the affinity of the sarco/endoplasmic reticulum Ca^2+^-ATPase (SERCA) for cytosolic Ca^2+^. Remarkably, and consistent with the phenotypic platform findings, computational modeling revealed that *PLN* KD [Michaelis constant (Km) of SERCA to cytosolic Ca^2+^ (forward pumping) (Kmf) 25%=75% reduction in PLN] caused a significant shortening of both APD_90_ and Ca^2+^ transient (Fig. 3E; Fig. S3E) in HAMs compared to control groups paced at 2 Hz. In conclusion, our multi-model system approach identifies *PLN* loss of function as a conserved and most potent hit driving APD shortening among the 20 AF-associated genes tested.

### *PLN* loss of function-induced arrhythmia depends on β-adrenergic pathway stimulation and co-culture with fibroblasts

**Fig. 4.**
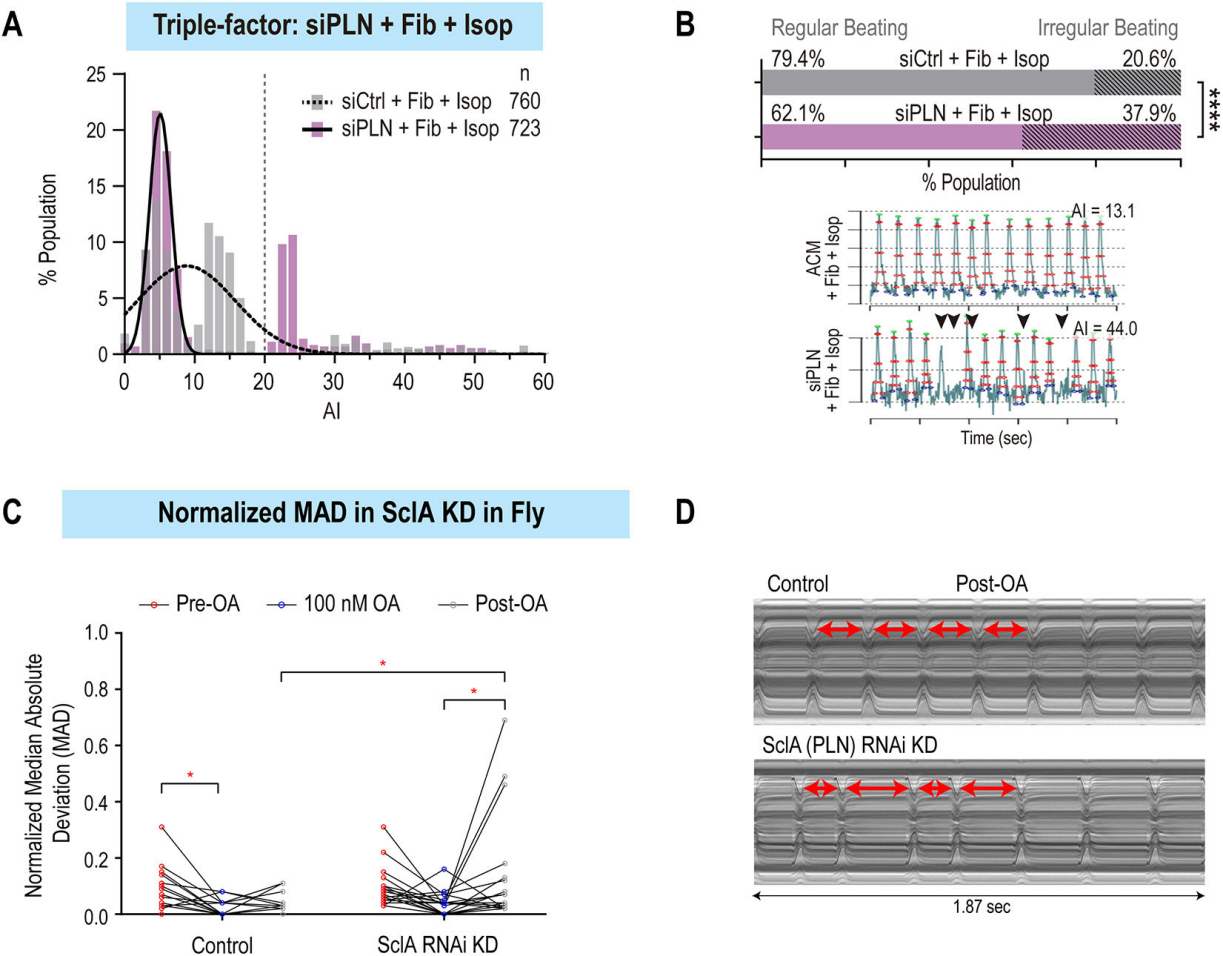
***PLN* KD in combination with environmental pertubagens induces arrhythmia phenotypes in ACMs and flies.** (A) Histogram of arrhythmia index (AI) values of ACMs co-cultured with fibroblasts and treated with Isop in siControl versus siPLN conditions. (B) Increased percentage of irregularly beating (AI>20) ACMs co-cultured with fibroblasts (Fib) and treated with Isop, in siPLN compared to siControl (top). Representative peak trains of APs show irregular beat-to-beat interval (black arrowheads) in siPLN compared to siControl condition (bottom). (C) Distribution of median absolute deviation (MAD) values before, during and after 100 nM octopamine (OA) treatment. Post-OA, *SclA* KD hearts exhibit increased arrhythmia compared to controls (*P<0.05, repeated measures two-way ANOVA). (D) Representative M-modes showing irregular beat-to-beat intervals in *SclA* KD hearts post-OA compared to control hearts (arrows show individual heart periods).

Next, to determine whether loss of *PLN* function alone is sufficient to induce arrhythmia-like phenotypes, we measured the beat-to-beat interval variance (AI) in ACMs upon *PLN* KD, and found no difference compared to siRNA that does not target any gene and serves as negative control (siControl) (Fig. S4A-D). Additional factors such as conductance heterogeneity due to atrial fibrosis (Dzeshka et al., 2015; Xintarakou et al., 2020) and sympathetic stresses (Workman, 2010) have been tightly linked with the onset and maintenance of AF, and are often categorized as ‘AF-associated risk factors’. Thus, to mimic these environmental perturbagens, we co-cultured ACMs with fibroblasts for 2[Supplementary-material sup1]days and/or applied the β-adrenoreceptor agonist, isoproterenol (1 µM), acutely upon kinetic imaging. Remarkably, co-culturing ACMs with fibroblasts in a 3:1 ratio nearly doubled the percentage of ACMs with AI values >20 (from 14.6% to 28.9%); however, in this context, *PLN* KD did not further increase the percentage of arrhythmic cells (Fig. S4D-F). Conversely, treating ACMs with isoproterenol alone did not increase the percentage of arrhythmic cells, whereas exposing ACMs to isoproterenol along with *PLN* KD increased the percentage of arrhythmic cells from 13% to 22% (Fig. S4G). Finally, co-culture with fibroblasts followed by acute isoproterenol treatment caused severe arrhythmia-like phenotypes (AI>20) in ∼38% of ACMs compared to 20% in the presence of perturbagens only (Fig. 4A,B). Interestingly, analysis of AP trains (lower panel in Fig. 4B) revealed missing beats and smaller refiring events that could be associated with DADs. Thus, collectively, our results indicate that reduced *PLN* function predisposes ACMs to arrhythmia upon sensitization by fibroblasts and acute β-adrenergic stimulation.

In fly hearts, despite significant changes in SI, neither AI nor MAD arrhythmia parameters were significantly altered by cardiac-specific *PLN*/*SclA* KD. To add an adrenergic stress, we exposed the fly hearts to octopamine (OA), the fruit fly version of norepinephrine/adrenaline (Sujkowski et al., 2017). Acute OA exposure significantly elevated heart rate by significantly shortening SIs in both control and KD lines, with a maximal effect at 100 nM, which was the dose used for subsequent pharmacological pacing of the fly heart (Fig. S5A,B). In controls, the mean SI returned to pre-exposure values at 10 min post-OA exposure (Fig. S5C), whereas that in *PLN*/*Sln* KD hearts did not (Fig. S5D). In addition to SI, contraction and relaxation intervals were significantly shortened in the presence of OA (Fig. S5E,F). We also observed increased post-OA pacing bouts of arrhythmia in the *PLN*/*Sln* KD hearts [mean normalized mean absolute deviation (nMAD)=0.1342; Fig. 4C,D], compared to hearts from controls (mean nMAD=0.0379; *P*=0.019, repeated measures two-way ANOVA).

### Computational modeling validates PLN as a key regulator of rhythm in human adult atria

**Fig. 5.**
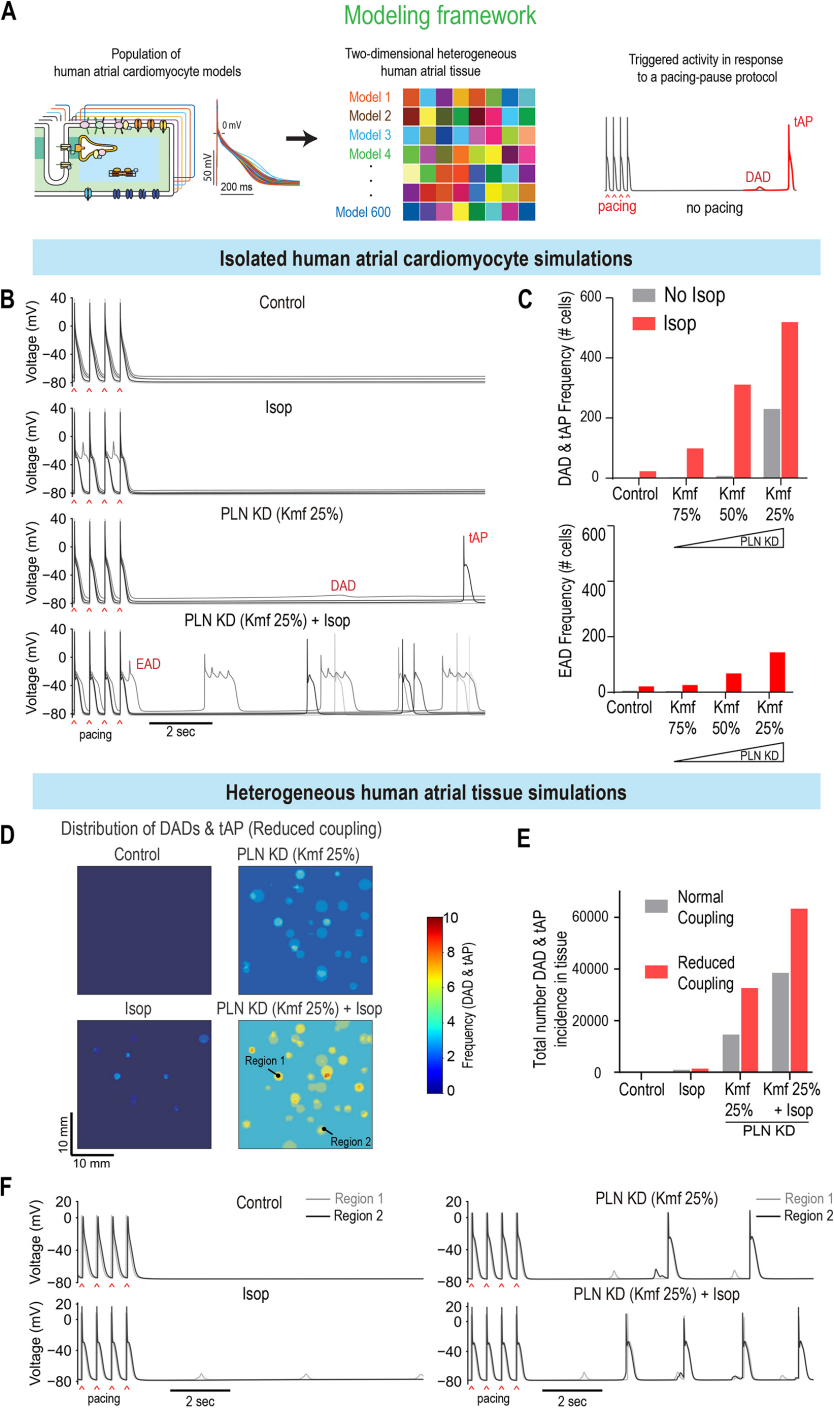
**Combined *PLN* KD and Isop challenge promotes arrhythmic events in isolated HAMs and two-dimensional (2D) atrial constructs.** (A) Modeling framework for evaluating arrhythmic events in HAMs and 2D human atrial tissue. Colors indicate that each cell from the population of computational models has distinct electrophysiological properties (i.e. AP waveforms) to mimic physiologic heterogeneity in cells. For the 2D model of human atrial tissue, each of the 600 cells is mapped into clusters, each of which has distinct properties compared to those of neighboring clusters. The physiological properties of each myocyte cluster were randomly assigned, thereby producing a heterogeneous tissue structure. A pacing (2 Hz)–pause protocol was applied to assess the incidence of triggered activities. DAD, delayed afterdepolarization; tAP, triggered AP. (B,C) Effects of *PLN* KD (Kmf 25%) and Isop on the triggered activity in human atrial CMs. (B) Time courses of APs for baseline (control), with Isop treatment, *PLN* KD (Kmf 25%), and combined Isop treatment and *PLN* KD (*PLN* KD+Isop). (C) Incidence of DAD and tAP (top), and EAD (bottom), detected in the HAM populations for Isop and various degrees of *PLN* KD (Kmf from 25% to 75%) conditions. (D-F) Effects of *PLN* KD (Kmf 25%) and Isop on the generation of triggered activity in heterogeneous human atrial tissue. (D) Spatial distribution of DADs and tAPs in the atrial tissue with reduced cell-to-cell coupling for *PLN* KD (Kmf 25%) and after Isop treatment. (E) Total number of DADs and tAPs detected in the atrial tissue after each perturbation with normal or reduced cell-to-cell coupling. (F) Superimposed traces of APs from two regions (marked in D) of the atrial tissue with reduced cell-to-cell coupling for each perturbation.

Next, to validate the phenotypic platform findings, we used models of isolated HAMs and two-dimensional atrial tissue, which allow modulation of cell-to-cell electrical coupling (Fig. 5A; Table S5; Colman et al., 2013; Ni et al., 2018), to account for the electrotonic effects of fibroblasts. In these assays, we applied a 2 Hz pacing–pause protocol to stimulate isolated HAMs or the left side of the atrial tissue construct and subsequently analyzed membrane voltage dynamics following the pause of pacing. We first tested the effect of increasing *PLN* KD on isolated HAMs (Kmf 25%=high KD; Kmf 75%=low KD) along with simulated isoproterenol treatment. Remarkably, increasing *PLN* KD levels led to the enhanced generation of triggered activity, which was further exacerbated by the simulated isoproterenol treatment (Fig. 5B,C). In this context, increasing *PLN* KD levels in combination with simulated isoproterenol treatment also led to an increase in the occurrence of EADs in isolated HAMs (Fig. 5C, bottom panel). At the tissue level, and similar to fibroblast co-culture with ACMs, *PLN* KD induced more triggered activity (DADs and triggered APs) when combined with treatment with isoproterenol and reduced cell-to-cell electrical coupling (Fig. 5D-F; Fig. S6A,B, Movies 1 and 2). Collectively, our findings suggest that *PLN* loss of function predisposes cells to arrhythmia in a tissue environment with reduced electrical coupling and elevated β-adrenergic activity.

### PLN functionally interacts with NCX and LTCCs to control rhythm

**Fig. 6.**
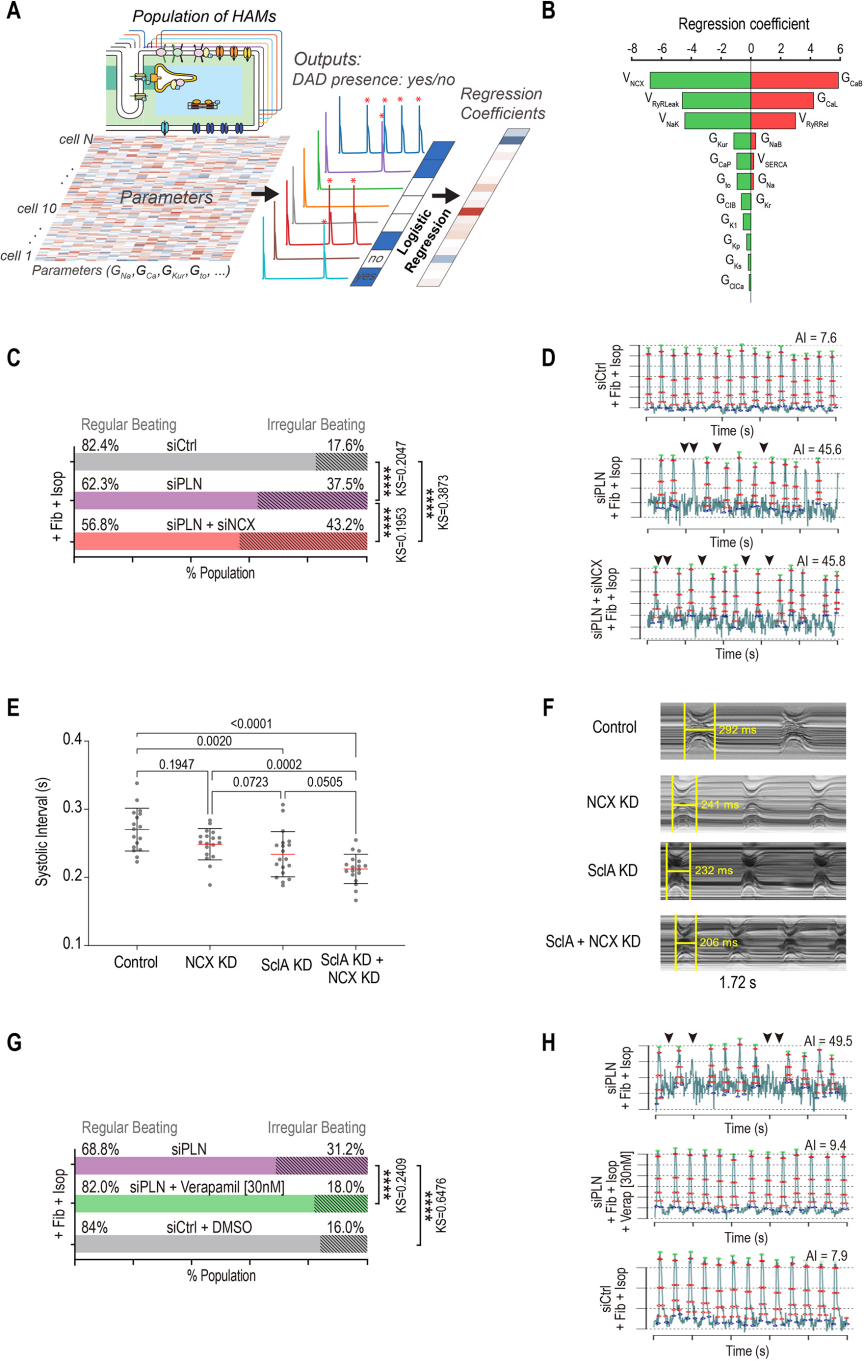
**Multiple perturbations are required to generate arrhythmicity across platforms.** (A) Schematic describing the logistic regression analysis approach to identify the mechanisms underlying the generation of DADs in HAMs. Red asterisks indicate the occurrence of DAD. G_Ca_, maximal conductance of L-type Ca^2+^ current; G_Kur_, maximal conductance of ultra-rapid delayed rectifier K^+^ current; G_Na_, maximal conductance of fast Na^+^ current; G_to_, maximal conductance of transient outward K^+^ current. (B) Logistic regression analysis of DAD incidence in the context of moderate *PLN* KD (Kmf 50%) revealed the influence of model parameters on the genesis of DADs in the population of HAMs in response to the pacing–pause protocol. Positive coefficients indicate that increasing the associated parameters promotes DAD production, and vice versa. G_CaB_, maximal conductance of background Ca^2+^ current; G_CaP_, maximal rate of plasma membrane Ca^2+^ ATPase current; G_ClB_, maximal conductance of background Cl^−^ current; G_ClCa_, maximal conductance of Ca^2+^-activated Cl^−^ current; G_Kp_, maximal conductance of plateau K^+^ current; G_Kr_, maximal conductance of rapid delayed rectifier K^+^ current; G_Ks_, maximal conductance of slow delayed rectifier K^+^ current; G_K1_, maximal conductance of inward rectifier K^+^ current; G_NaB_, maximal conductance of background Na^+^ current; V_NaK_, maximal pump rate of Na^+^/K^+^ pump current; V_NCX_, maximal exchange rate of Na^+^/Ca^2+^ exchange current; V_RyRLeak_, maximal rate of ryanodine receptor Ca^2+^ leak; V_RyRRel_, maximal rate of ryanodine receptor Ca^2+^ release; V_SERCA_, maximal rate of sarcoplasmic reticulum Ca^2+^ ATPase flux. (C) The percentage of irregularly beating (AI>20) ACMs co-cultured with fibroblasts and treated with Isop is increased when transfected with siPLN and siNCX compared to siPLN alone. (D) Representative AP peak trains for siControl, PLN siPLN+siNCX conditions in ACMs co-cultured with fibroblasts and treated with Isop. Arrowheads show examples of irregular beat-to-beat intervals in arrhythmically beating ACMs. (E) Mean SI in response to cardiac KD of the plasma membrane Na^+^/Ca^2+^ exchanger *NCX*, *SclA*, and combined *SclA*+*NCX* KD. Co-KD caused a greater decrease in SI than did single KD alone (Wilcoxon ranked sum test). (F) Representative M-modes showing the effects of KD on SI. (G) The percentage of irregularly beating (AI>20) ACMs co-cultured with fibroblasts and treated with Isop is decreased with verapamil (30 nM) treatment in comparison to that with DMSO treatment. (H) Representative AP peak trains and AI values for siPLN; siPLN+verapamil (30 nM); siControl conditions in ACMs co-cultured with fibroblasts and treated with Isop. Arrowheads show examples of irregular beat-to-beat intervals in arrhythmically beating ACMs. ****P<0.0001 (KS-D).

To characterize how PLN controls rhythm homeostasis in atrial myocytes, we observed that, at Kmf 50%, only half of HAMs generated DADs (see Fig. 5C). Thus, to uncover mechanisms underlying DAD generation in HAMs at Kmf 50%, we separated DAD-generating HAMs from non-DAD-generating HAMs and applied logistic regression analysis (Morotti and Grandi, 2017) to describe the relationship between model parameters and DAD incidence (Fig. 6A; Table S4). This analysis predicted that increased background Ca^2+^ current (I_CaB_), L-type Ca^2+^ current (I_CaL_), or RyR release flux [i.e. by augmenting the parameters maximal conductance of ICaB (G_CaB_), maximal conductance of ICaL (G_CaL_) or maximal flux rate for RyR release (V_RyRRel_)] in HAMs would promote the propensity for developing DADs. Similarly, our model also predicted that DAD occurrence correlated with reduced function of NCX, RyR leakiness, Na^+^–K^+^ pump NaK, or ultra-rapid delayed rectifier K^+^ current (I_Kur_) (Fig. 6B).

To validate these predictions, we selected two parameters that were most positively (I_CaL_ conductance) or negatively (NCX maximal transport rate) correlated with DAD incidence. First, we tested in ACMs whether reduced expression of the Na^+^–Ca^2+^ exchanger NCX, in the background of *PLN* KD would further increase the percentage of arrhythmic cells. Consistent with the model prediction, combined KD of *PLN* and *NCX* in the presence of perturbagens (fibroblasts co-culture and isoproterenol infusion) significantly increased the occurrence of arrhythmia-like phenotypes in ACMs compared to that after single *PLN* KD, from 37.5% to 43.2% of cells with AI>20 (Fig. 6C). Notably, an increase in arrhythmic phenotypes was coincident with the generation of short APs in siPLN/NCX condition compared to siPLN alone (Fig. 6D). To determine whether the interaction between PLN and NCX is conserved at the whole-heart level, we performed single KD and co-KD of *NCX*/*Calx* and *PLN*/*SclA* using the Hand4.2-Gal4 heart-specific driver line flies. Remarkably, although median SI was reduced in response to *PLN*/*SclA* KD (232 ms) or *NCX*/*Calx* KD (253 ms, Fig. 6E,F), KD of both genes further shortened the SI median to 211 ms (*P*=0.05, Wilcoxon ranked sum test).

Finally, the regression analysis also revealed that DAD-generating HAMs had increased LTCC (G_CaL_) currents. Thus, to test whether inhibition of LTCC activity might reduce PLN-induced arrhythmia phenotypes, we treated ACMs with a Ca^2+^ channel blocker, verapamil, and quantified the percentage of arrhythmic cells in response to *PLN* KD+co-culture with fibroblasts+isoproterenol treatment. Remarkably, ACMs treated with verapamil were 1.7-fold less arrhythmic than those treated with dimethyl sulfoxide (DMSO) control (from 31.2% to 18%; Fig. 6G,H). Collectively, our results indicate that *PLN* KD-dependent arrhythmia phenotypes are, at least in part, mediated by NCX and LTCC activity. Our results also highlight that the combined use of computational modeling and our phenotypic platform represents an effective approach to identify gene interactions involved in the regulation of atrial rhythm and to potentially predict potential therapeutic targets to treat AF.

## DISCUSSION

### Multi-system platform to model AF

**Fig. 7.**
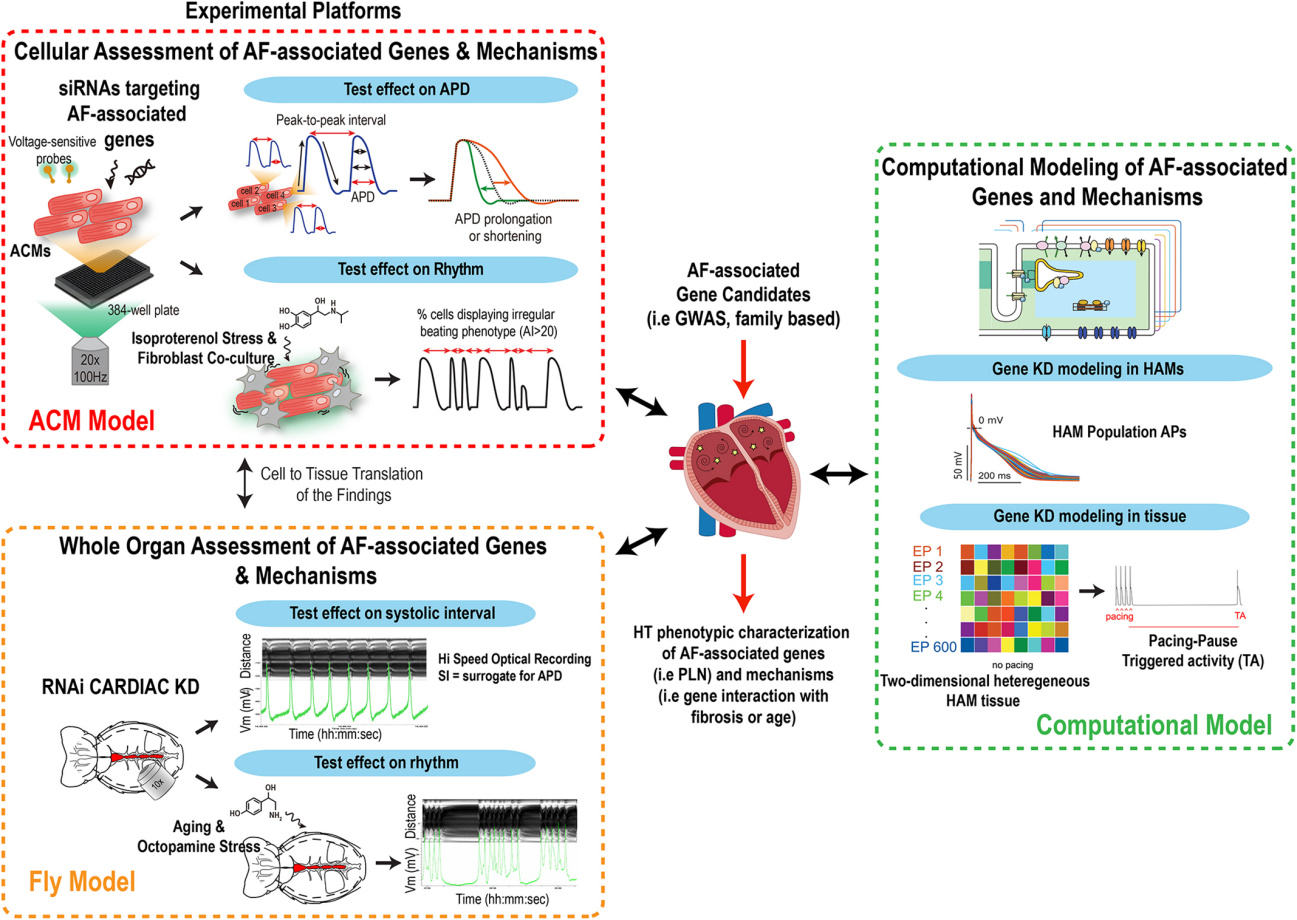
**Novel multiplatform modeling of AF.** Schematic summarizing how our integrated multiplatform approach enables the HT identification and characterization of AF-associated genes and mechanisms, using model systems with human, adult, whole-organ and atrial relevance. GWAS, genome-wide association studies.

As for other common cardiovascular diseases (i.e. hypertension, myocardial infarction), AF is caused by complex and mostly unknown combinations of genetic (Roselli et al., 2020) and environmental (i.e. fibrosis, age) (Schuettler) insults that render experimental modeling a challenging process. In this context, and due to the growing list of AF-associated genes (Nielsen et al., 2018a; Roselli et al., 2018), we assessed that an ideal modeling strategy should include the ability to rapidly test the effects of a large cohort of genes on cardiac electrophysiological and rhythm parameters, along with their interaction with other genes and/or environmental perturbagens (i.e. age, fibrosis or hormonal stress), preferably in a human adult atrial context. To address some of these modeling requirements, we have assembled (Schuttler et al., 2020) a new phenotypic platform that enables rapid evaluation of the effects of gene function on the regulation of APD and rhythm in model systems, providing (1) a human and atrial context (ACM model), and (2) an intact, functionally mature and aging heart (fly model). Because both ACM and fly systems have genome-wide screening capacity (Neely et al., 2010; Nielsen et al., 2022 preprint), they unlock the exploratory power of functional genomics that is needed for the unbiased identification of novel genes and pathways controlling cardiac rhythm beyond those identified in GWAS. A remarkable feature of the ACM platform is the ability to assess APD and rhythm parameters at single-cell resolution, which enables (1) the development of co-culture conditions mimicking aspects of known AF-associated risk factors, such as fibrosis, and (2) the quantification of the heterogeneity in cellular responses to environmental perturbations. Similarly, in flies, rapid aging (a 5-week-old fly is considered an old fly) (Blice-Baum et al., 2019; Ocorr et al., 2007b) and the ability to expose these animals to environmental stressors, such as OA or different diets (high fat or high sugar) (Birse et al., 2010; Johnson et al., 1997), enables the study of genes' functions and their interaction with the environment in a physiologically integrated heart system. Because ACMs represent a relatively immature state of adult HAMs (Uosaki et al., 2015) and the fly heart architecture significantly differs from that of mammals, findings using these platforms need to be further validated in models with human adult atrial relevance. Thus, to address these limitations, we have incorporated computational models of adult HAMs as a third model and a tool for both validation (Figs 3 and 5) and hypothesis generation (Fig. 6). In sum, we propose that the integrated use of model systems combining functional screening capacity and human atrial and whole-organ physiological relevance represents a novel approach to enable the identification and characterization of new genes affecting AF-associated rhythm biology with unprecedented throughput (Fig. 7).

### Cellular arrhythmia is a compound phenotype caused by multiple perturbagens

Cellular arrhythmias associated with AF are known to arise from ‘vulnerable substrates’ caused by APD prolongation or shortening, and thereby promoting EADs and/or DADs or circuit re-entry, respectively. In this context, conductance heterogeneity due to atrial fibrosis, as well as sympathetic stresses, have been tightly linked with the onset of AF and are often categorized as ‘AF-associated risk factors’ (Fatkin et al., 2017). Here, we have replicated the effects of some of these risk factors (isoproterenol and/or fibroblasts in ACMs and OA in the fly heart) and observed that multiple stressors were required to produce robust arrhythmia phenotypes upon AF-associated gene KD (*PLN*). These results also emphasize the requirement for model systems that permit the incorporation of such risk factors for efficient arrhythmia modeling. In this context, a remarkable contribution of the fly model is the observation that cardiac arrhythmias can occur in response to ion channel knockout and KD (Ocorr et al., 2007c, 2017). Interestingly, these arrhythmias only became robust with increasing fly age, and, in the case of the KCNQ channel, this was linked to an age-related reduction in channel expression that could be reversed by channel overexpression in old flies (Nishimura et al., 2011). In sum, these new models facilitate the molecular delineation of how environmental insults synergize with genetic predispositions to induce arrhythmia-associated phenotypes and thus represent a promising new research direction to uncover novel therapeutic avenues to treat AF.

### Mechanism of *PLN*-driven arrhythmia in atrial myocytes

In this study, we identified *PLN* loss of function as the top hit, among the 20 AF-associated genes tested, causing both APD shortening and arrhythmia phenotypes (i.e. beat-to-beat interval irregularities, DADs) when combined with environmental perturbagens. Consistent with a potential role for *PLN* as a AF-contributing gene, three large independent GWAS (Christophersen et al., 2017; Nielsen et al., 2018a; Roselli et al., 2018) have identified SNPs in the vicinity of the *PLN* gene locus in patients with AF (Christophersen et al., 2017; Nielsen et al., 2018a; Roselli et al., 2018), although the functional significance of such variants is unknown. Mechanistically, our simulations in HAMs suggest that *PLN* loss of function selectively increases atrial SERCA pump activity and the sarcoplasmic reticulum Ca^2+^ load while shortening CTD. The associated Ca^2+^ overload and spontaneous DADs, in turn, contribute to cause electrical instabilities. Consistent with our model, increased SERCA function is observed during paroxysmal AF (Denham et al., 2018; Voigt et al., 2014) and overexpression of SERCA in mouse atria promotes cellular correlates of AF (Nassal et al., 2015). Interestingly, similar results have also been reported with the ablation of the *PLN* functional homolog, sarcolipin, where marked structural and electrical atrial remodeling was reported in mice (Babu et al., 2007). Furthermore, our computational modeling approach also indicated that *PLN* KD-induced arrythmia phenotypes are exacerbated by increased L-type Ca^2+^ current and decreased NCX activity. These simulations suggest that LTCCs and NCX modify PLN activity and contribute to regulate rhythm in atrial myocytes, and thus they might represent a class of second hits contributing to promote *PLN* KD-induced arrythmias. Consistent with these predictions, co-KD of *PLN* and *NCX* in ACMs increased the percentage of cells displaying arrhythmic phenotypes, whereas inhibition of LTCC activity markedly reduced these phenotypes. In the fly heart, interactions between these same two genes were also pro-arrhythmogenic in that co-KD significantly shortened the SI relative to individual gene KD. Collectively, these observations suggest a central role for the PLN–SERCA–LTCC–NCX axis in the regulation of AF-associated rhythm phenotypes, which is consistent with the mechanisms known to contribute to AF pathophysiology (reviewed in Denham et al., 2018), and thereby highlight the physiological relevance of this new approach to model AF.

### What's next – next steps

This platform provides an in-depth resolution of cardiac electrophysiology metrics with various applications: (1) large-scale functional genomic screens to identify novel gene regulatory networks governing cardiac rhythm; (2) establishment of new arrhythmia models to phenotypically characterize rhythm-associated cardiac diseases; and (3) small-molecule screens for anti-arrhythmic drug discovery.

### Limitations of the system

Limitations of the presented platform include the use of hiPSC-derived ACMs to study AF, a known age-dependent disease. Although CM differentiation techniques are advancing, single-cell RNA-seq results from various studies implied that hiPSC-derived CMs are in similar transcriptional stages to those of pre-neonatal or fetal CMs (DeLaughter et al., 2016; Friedman et al., 2018). Physiological differences, such as cellular morphometry, functional maturity, transcription profile and disease manifestation, of these relatively immature hiPSC-derived ACMs may undermine the use of the platform to study a highly age-dependent disease. To mimic the conductance heterogeneity due to cardiac fibrosis, fibroblasts were co-cultured with ACMs in a monolayer format. This simplified reproduction of *in vitro* cardiac fibrosis model may not be sufficient to fully recapitulate complex arrhythmia mechanisms that occur at the organ level. Such limitation could be overcome with the advancement of current culturing technics, including micropatterning cell culture substrates or cardiac organoids (Salick et al., 2014; Zhao et al., 2021). The lack of stimulus apparatus of the platform restricts certain pacing-induced arrhythmia studies. However, incorporating compounds with beat rate-regulating properties, such as β-adrenergic modulators, may provide an alternative approach for bradycardia/tachycardia-induced arrhythmias. Lastly, the siRNA KD approach applied in this study is limited in evaluating the loss of function of the AF-associated gene candidates. Previous studies indicate that the gain-of-function mutation (i.e. KCNA5) and the abnormally elevated expression level (i.e. PITX2) can contribute to the initiation and maintenance of AF (Christophersen et al., 2013; Syeda et al., 2016). This can easily be tested in the *Drosophila* model, in which tissue-specific gene overexpression is achieved using the same Gal4 driver system used for gene KD. We are currently developing an ACM screening assay that incorporates a CRISPR-activation technique for gain-of-function mutation screens (Heidersbach et al., 2023).

## MATERIALS AND METHODS

### Generation of ACMs

hiPSCs were derived from dermal fibroblasts and donated by the laboratory of Dr Joseph Wu (Stanford University, CA, USA). Id1-overexpressing hiPSCs were dissociated with 0.5 mM EDTA (Thermo Fisher Scientific) in PBS without CaCl_2_ and MgCl_2_ (Corning) for 7 min at room temperature. hiPSCs were resuspended in mTeSR-1 medium (StemCell Technologies) supplemented with 2 µM thiazovivin (StemCell Technologies) and plated in a Matrigel-coated 12-well plate at a density of 3×10^5^ cells per well. Twenty-four hours after passage, cells were fed daily with mTeSR-1 medium (without thiazovivin) for 3-5 days until they reached ≥90% confluence to begin differentiation. hiPSC-derived ACMs were differentiated as previously described. At day 0, cells were treated with 6 µM CHIR99021 (Selleck Chemicals) in S12 medium for 48 h. At day 2, cells were treated with 2 µM Wnt-C59 (Selleck Chemicals), an inhibitor of the WNT pathway, in S12 medium. Forty-eight hours later (at day 4), the S12 medium was fully changed. At day 5, cells were dissociated with TrypLE Express (Gibco) for 2 min and blocked with RPMI (Gibco) supplemented with 10% fetal bovine serum (FBS; Omega Scientific). Cells were resuspended in S12 medium supplemented with 4 mg/l Recombinant Human Insulin (Gibco) (S12+ medium), 300 nM retinoic acid (R2625-50MG) and 2 µM thiazovivin and plated onto a Matrigel-coated 12-well plate at a density of 9×10^5^ cells per well. The S12+ medium was changed at day 8 and replaced at day 10 with RPMI (Gibco) medium supplemented with 213 µg/µl L-ascorbic acid (Sigma-Aldrich), 500 mg/l BSA-FV (Gibco), 0.5 mM L-carnitine (Sigma-Aldrich) and 8 g/l AlbuMAX Lipid-Rich BSA (Gibco) (CM medium). Typically, hiPSC-derived ACMs start to beat at ∼day 9-10. At day 15, cells were purified with lactate medium [RPMI without glucose, 213 µg/µl L-ascorbic acid, 500 mg/L BSA-FV and 8 mM sodium-DL-lactate (Sigma-Aldrich)], for 4 days. At day 19, the medium was replaced with CM medium.

### Voltage assay in ACMs

Voltage assay was performed using the labeling protocol described in McKeithan et al. (2017). Briefly, hiPSC-derived ACMs at day 25 of differentiation were dissociated with TrypLE Select 10X (Thermo Fisher Scientific) for up to 10 min, and the action of TrypLE Select 10X was neutralized with RPMI supplemented with 10% FBS. Cells were resuspended in RPMI with 2% KOSR (Gibco) and 2% B27 50X with vitamin A (Life Technologies) supplemented with 2 µM thiazovivin and plated at a density of 6000 cells per well in a Matrigel-coated 384-well plate. hiPSC-derived ACMs were then transfected with siRNAs directed against AF-associated genes (ON-TARGETplus Human, Horizon Discovery; siGATA4, J-008244-05-0002; siGATA5, J-010324-06-0005; siGATA6, J-008351-06-0005; siGJA1, J-011042-05-0002; siGJA5, J-017368-05-0002; siHAND2, J-008698-06-0005; siHCN4, J-006203-05-0002; siKCNA5, J-006215-06-0005; siKCND3, L-006226-00-0005; siKCNJ5, J-006250-06-0002; siKCNN3, J-006270-06-0002; siNKX2-5, J-019795-07-0002; siNKX2-6, J-025793-17-0002; siNPPA, J-012729-05-0002; siPITX2, J-017315-05-0005; siPLN, J-011754-05-0005; siSH3PXD2A, J-006657-07-0002; siSYNE2, J-019259-09-0002; siTBX5, J-013410-05-0002; siZFHX3, J-015412-05-0002) using lipofectamine RNAi Max (Thermo Fisher Scientific). Each siRNA was tested in biological quadruplicates for each differentiation, and differences between experimental conditions and controls were replicated in at least two independent differentiations. Every 3 days post-transfection, cells were first washed with pre-warmed Tyrode's solution (Sigma-Aldrich) by removing 50 µl medium and adding 50 µl Tyrode's solution five times using a 16-channel pipette. After the fifth wash, 50 µl of 2× dye solution consisting of voltage-sensitive dye Vf2.1.Cl (Fluovolt, Thermo Fisher Scientific; 1:2000) diluted in Tyrode's solution supplemented with 1 µl of 10% Pluronic F127 (diluted in water; Thermo Fisher Scientific) and 20 µg/ml Hoescht 33258 (diluted in water; Thermo Fisher Scientific) was added to each well. The plate was placed back in the 37°C 5% CO_2_ incubator for 45 min. After the incubation time, cells were washed four times with fresh pre-warmed Tyrode's solution using the same method described above. hiPSC-derived ACMs were then automatically imaged with an ImageXpress Micro XLS microscope at an acquisition frequency of 100 Hz for a duration of 5 s with an excitation wavelength of 485/20 nm and emission filter 525/30 nm. A single image of Hoescht 33258 was acquired before the time series. Fluorescence over time quantification and trace analysis were performed using custom software packages developed by Molecular Devices and the A.R.C. laboratory. Although, cells were not paced during the APD measurement process, beat rate was controlled *in silico* by only comparing APDs between conditions in which peak trains had similar beat rate (±10%), thereby minimizing the effect of beat rate on APD.

### Arrhythmia assay and drug treatment in ACMs

hiPSC-derived ACMs were dissociated, plated in a 384-well plate and transfected with siRNAs targeting AF-associated genes (ON-TARGETplus Human; siNCX, J-007620-05-0002). Twenty-four hours post-transfection, 2000 primary human fibroblasts per well were added to the hiPSC-derived ACMs. Forty-eight hours later (the day of the imaging), cells were dyed with the voltage-sensitive dye Vf2.1.Cl as described above, then treated with 50 µl of a 2× solution of isoproterenol (1 µM final) diluted in Tyrode’s solution alone and in combination with a 2× solution of verapamil (30 nM final), diluted in Tyrode’s solution, at the fifth wash. After 20 min of compound incubation time, cells were imaged, and single-cell traces were analyzed as described previously.

### Whole-cell patch-clamp electrophysiology

Cardiac ion currents were recorded from single CMs using the whole-cell patch-clamp method. Briefly, coverslips with ACMs or VCMs were transferred into an electrophysiologically perfused recording chamber (RC-25-F, Warner Instruments, Hamden, CT, USA) mounted on the stage of an inverted Olympus microscope. Patch pipettes were pulled from thin-wall borosilicate glass capillaries (CORNING 7740, 1.65 mm) with a P-2000 laser pipette puller (Sutter Instruments, Novato, CA, USA) and had electrode tip resistances between 1.5 and 5.5 MΩ with access resistance of <8 MΩ for whole-cell patch recordings. Series resistance and cell capacitance were compensated to between 30% and 60% in some voltage-clamp recordings. For current-clamp recordings, pipettes contained the following: 76 mM potassium aspartate, 20 mM KCl, 2.5 mM MgCl, 10 mM HEPES, 4 mM NaCl, 6 mM CaCl_2_, 10 mM K4EGTA, 5 mM K2ATP and 0.1 mM Na-GTP (pH 7.2; 310 mOsm). All recordings were collected at room temperature in Tyrode's solution. Current response traces were acquired using an Axon 200B amplifier. Currents were digitally sampled at 10 kHz using Digidata 1322A digitizer hardware and pClamp 10.2 software (Molecular Devices). For ACMs and VCMs, *n*=5.

### *Drosophila* strains

We used the Hand4.2-Gal4 fly line as our heart-specific driver line (Brand and Perrimon, 1993). Virgin Hand4.2-Gal4 females were crossed to male flies from UAS-RNAi lines for each AF gene candidate. UAS-RNAi lines and their respective control lines were acquired from the Bloomington *Drosophila* Stock Center (BDSC; Bloomington, IN, USA) and Vienna *Drosophila* Resource Center (VDRC; Vienna, Austria). For each gene candidate, at least two different RNAi lines were used (Table S6; GD and KK were the genetic background lines for stocks from VDRC, and ATTP2 and ATTP40 were the genetic background lines for stocks from BDSC).

The *PLN* (fly ortholog *SclA*)-sensitized fly line was made by recombining the USA-SclA RNAi with the Hand4.2-Gal4 heart-specific driver line. Virgin females from the Hand4.2-Gal4 or the *SclA*-sensitized, Hand4.2-Gal4 driver lines were crossed to males of the desired UAS-RNAi lines. Adult female flies for all crosses were collected upon eclosion and raised at 25°C on a 12 h light–dark cycle. Flies were fed a standard yeast–cornmeal diet, with food replaced every other day.

### *Drosophila* heart function characterization

Cardiac phenotypes of middle-aged (3[Supplementary-material sup1]weeks old) female flies from each cross were characterized using denervated, semi-intact preparations as previously described (Ocorr et al., 2009; Vogler and Ocorr, 2009). Briefly, hearts from 20-25 flies were examined for each genotype and age. Adult female flies were exposed to FlyNap (Carolina Biological Supply), a triethylamine-based anesthetic, for at least 1 min until no movement was detected. Hearts were exposed by dissection in room temperature, air bubbled, artificial hemolymph (AHL; Ocorr et al., 2007a,c). High-speed video recordings were filmed with a Hamamatsu EM-CCD camera and using HC Image capture software (Hamamatsu). Heart movements were analyzed using the Semi-automated Optical Heartbeat Analysis (SOHA) software. Movies were recorded at speeds of 140+ fps with pixel resolution of ∼1 μm/pixel, allowing very precise temporal and spatial measurements, including heart period (HP) and rate (1/HP), diastolic interval and SI, and fractional shortening/contractility. To quantitate arrhythmia, we first calculated the MAD. The median value of the absolute deviations of each heart period (*X*_i_) from the median heart period 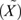 was calculated and then multiplied by a constant (*k*=1.4826 assuming data are normally distributed):
(1)

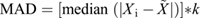


To normalize the MAD index (nMAD), the MAD value was divided by the median heart period. Qualitative records of heart wall movements (M-modes/kymographs) were produced by electronically excising a 1 pixel horizontal ‘slice’ from each movie frame and aligning them horizontally, providing an edge trace displaying heart wall movements in the *x*-axis over time along the *y*-axis (Ocorr et al., 2009, 2007c).

### OA-challenge heart assay

OA pacing experiments were performed *in situ* on the semi-intact fly preparation. OA (Sigma-Aldrich, O0250) stock solution (10 mM) was freshly prepared by dissolving in water and was further diluted in AHL. A dose–response curve was generated using doses ranging from 0.1 nM OA to 500 nM OA (Fig. S5A). The increase in heart rate was maximal at 100 nM OA, which was the dose used for all subsequent pacing experiments (Fig. S5B). Following dissection, hearts were first allowed to equilibrate in fresh AHL for 15 min, and 30 s movies of heart function were recorded. Heart function was recorded three times per fly: (1) pre-drug exposure, (2) after a 15-min exposure to 100 nM OA, and (3) after a 15 min post-drug exposure recovery period. A second set of hearts exposed only to vehicle (AHL) were filmed at the same three 15 min intervals to serve as time controls.

### Simultaneous optical and electrophysiological recordings

Simultaneous optical and intracellular electrical recordings were performed as previously described (Ocorr et al., 2017). Briefly, we used a semi-intact preparation that was incubated in AHL. Optical recordings were done as described above; electrical potentials were recorded using sharp glass electrodes (20±50 MΩ) filled with 3 M KCl and standard intracellular electrophysiological techniques. Data were acquired using an Axon-700B Multiclamp amplifier, signals were digitized using the DIGIDATA 1322A, and data were captured and analyzed using PClamp 9.0 and Clampfit 10.0 software, respectively (all from Molecular Devices). Data were quantified from representative 30 s recordings in which the resting membrane potential had remained stable for at least 30 s. To coordinate the optical and electrical recordings, a transistor-transitor logic (TTL) pulse was sent by the image capture software to the Digitizer. The pulse duration lasted for the entire period of optical recording and was recorded in a separate channel by the PClamp software, allowing us to delineate the beginning and the end of the optical recording and directly align it with the electrical record.

### Statistical analysis

#### ACMs

Population distribution of control and siRNA-treated hiPSC-derived ACMs was generated with GraphPad Prism software (2019) using nonlinear regression. Unpaired nonparametric KS-D was used to compare each treated condition to control using APD_75_ of every measured cell. To determine any statistical significance between experimental and control groups, we calculated two-sided *P*-values with paired two-tailed Student's *t*-test using GraphPad Prism software.

#### Flies

Data that exhibited a normal distribution (Shapiro–Wilk test) were evaluated for significance using a one-way ANOVA (for simple comparisons) or a two-way ANOVA (for multiple manipulations) followed by Sidak's multiple comparisons post-hoc tests as indicated in figure legends. Datasets that did not show a normal distribution (typically, heart period, SI and diastolic interval, and arrhythmia parameters) were analyzed using a nonparametric Wilcoxon rank sum test or Kruskal–Wallis test followed by Dunn multiple comparisons post-hoc tests. For acute OA stress experiments, we used repeated measures two-way ANOVA with a Greenhouse–Geisser correction to address potential lack of sphericity, followed by Sidak's multiple comparisons test. If data did not meet assumptions of normality, we log transformed our data and repeated the repeated measures two-way ANOVA. Statistical analysis and data visualization were completed with GraphPad Prism (v8.0.0), R (v3.6) and Rstudio (v1.3.959).

### Computational modeling design

We employed our well-established computational model (Grandi et al., 2011) of HAMs to simulate human AP and Ca^2+^. PLN regulates SERCA function by decreasing the apparent affinity of SERCA for Ca^2+^ ions (Periasamy et al., 2008; Simmerman and Jones, 1998). Accordingly, the effects of *PLN* KD on SERCA were simulated by various degrees of reduction in the SERCA affinity parameter (Kmf) for cytosolic Ca^2+^: Kmf was scaled by 75%, 50% or 25% to cover a wide parameter space of change. These changes were made based on a previous study showing that applying anti-PLN antibody shifted the affinity from 0.8 μM to 0.2 μM (Cantilina et al., 1993).

### Modeling arrhythmias in human atrial cells

To describe the intrinsic cell-to-cell variabilities in atrial electrophysiology and uncover the uncertainty of the modeling results, we applied a population-based approach (Ni et al., 2018; Sobie, 2009) to build populations of 600 human atrial model variants by randomly perturbing key model parameters (e.g. the maximum ion channel conductances, rates for membrane transporters, and Ca^2+^ handling fluxes; detailed in Table 4) by a lognormal distribution (σ=0.2).

### Logistic regression analysis of DADs

We performed logistic regression analysis (Morotti et al., 2017) to understand the influence of each model parameter on the arrhythmic outcome in HAMs. For each cell of the population of models, a binary code (yes/no) was applied to describe the presence/absence of DADs. Logistic regression coefficients were obtained using MATLAB (R2019b) scripts as detailed previously (Morotti and Grandi, 2017).

### Modeling arrhythmias in human atrial tissue

We created two-dimensional (2D) models to understand the dynamic behaviors of atrial AP and Ca^2+^ in tissue using a monodomain equation (Clayton et al., 2011) to describe the tissue electrical coupling as we did in our previous studies (Ni et al., 2017):
(2)

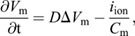
where *V*_m_ is the membrane potential of CMs, *i*_ion_ represents total ionic current, *C*_m_ is the capacitance of the cell membrane, and *D* indicates the isotropic diffusion coefficient describing the cell-to-cell coupling strength. The 2D model comprises 120×125 grids with a spatial interval of 0.25 mm. To account for the intrinsic variabilities in tissue, we mapped our population of 600 models to the tissue based on a heterogeneous pattern, dividing the tissue into 600 blocks consisting of 5×5 grids. Under normal coupling, we set *D* as 0.1485 mm^2^/ms, so that the conduction velocity under normal conditions is aligned with previous experimental observations and consistent with modeling studies (Aslanidi et al., 2011; Colman et al., 2013; Harrild and Henriquez, 2000; Heida et al., 2021; Konings et al., 1994). To assess how tissue coupling affects the arrhythmic events, we also simulated a reduced coupling (scale to 25% of tissue conductivity) condition. The resulting conduction velocity with normal or reduced tissue coupling is provided in Table S5. In agreement with previous experimental and modeling observations (Lachaud et al., 2022), our simulations showed that the APD variations seen at the single-cell level are reduced in coupled tissue (Fig. S7), and these are associated with the strength of tissue coupling: increasing the cell-to-cell coupling further reduces the APD variation.

### Pacing–pause protocol in single-cell and tissue stimulation

A constant pacing–pause protocol was applied to evaluate the physiological effects of *PLN* KD. Specifically, single cells were paced at 2 Hz for 290 s prior to a 10 s period of pause without stimulation. In tissue simulations, stimuli were applied on the left side of the 2D tissue at 2 Hz for 10 s, which was followed by a 10 s period without stimulation. AP and Ca^2+^ traces from the last four stimuli and the non-paced period were recorded for data analysis. Logistic regression analysis was applied to uncover the influence of model parameters on the incidence of arrhythmogenic events.

## Supplementary Material

10.1242/dmm.049962_sup1 Supplementary information
