# Supplementary material for: Multiplatform modeling of atrial fibrillation identifies phospholamban as a central regulator of cardiac rhythm
Source: Dis Model Mech. 2023 Jul 17;16(7):dmm049962. doi: 10.1242/dmm.049962 (PMC10387351; doi:10.1242/dmm.049962)
Supplement: Supplementary information [file dmm-16-049962-s1.pdf]

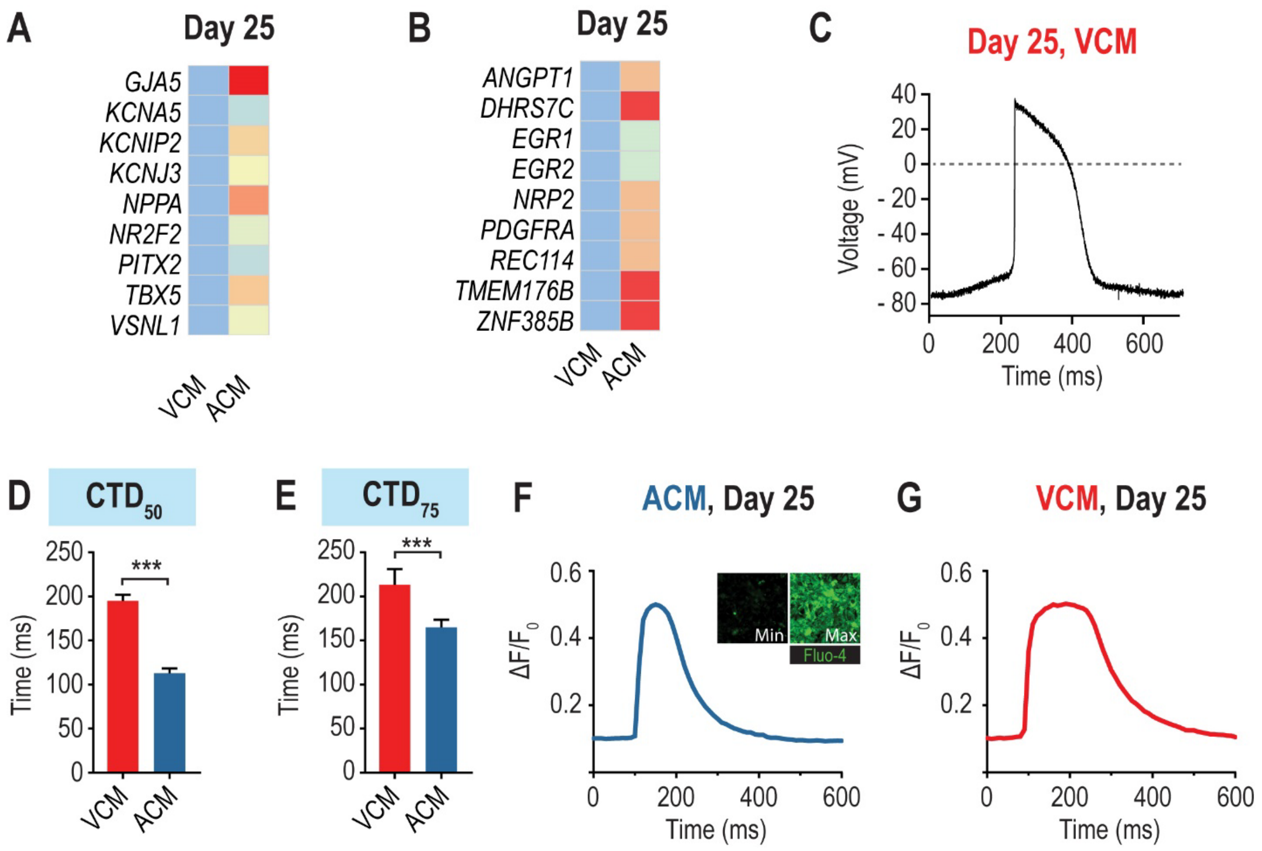

Fig. S1. Gene expression and functional characterization of ACMs. (A & B) Heatmap of atrial genes enriched in day 25 ACMs as compared to VCMs. **(C)** Patch- clamp experiments show that VCMs generate action potential with ventricular characteristics, including a long phase 2. **(D,E)** Quantification of average CTD50 and CTD75 demonstrate that ACMs display shorter calcium transients than VCMs. P-value *** < 0.001. **(F,G)** Representative calcium transient traces for ACMs and VCMs respectively.

Disease Models & Mechanisms • Supplementary information

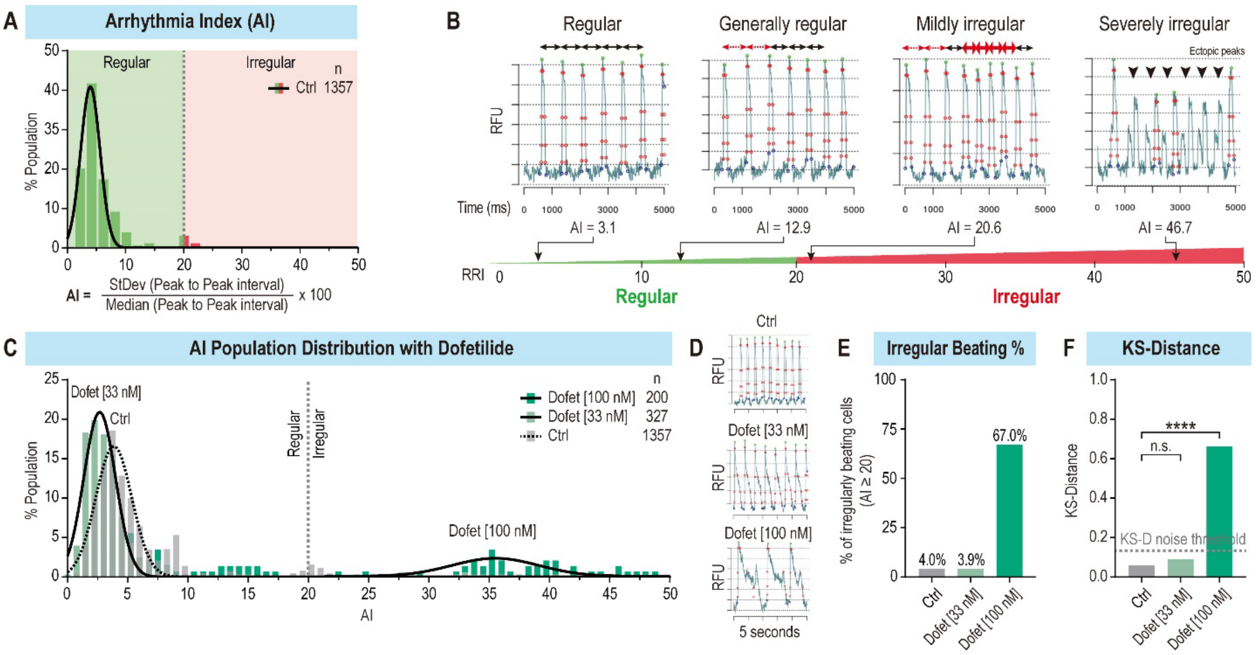

Fig. S2. Quantification of rhythm parameters in ACMs. (A) Example of histogram with Arrhythmia Index (AI) values showing cells with AI<20: not arrhythmic or AI > 20 arrhythmic. **(B)** Examples of peak trains from ACMs with regular (left), mildly irregular (middle), and severely irregular (right) with their respective AI score. **(C)** Histogram representing the distribution of AI values for ACMs treated with increasing doses of Dofetilide. **(D)** Representative peak trains of control ACMs (top), ACMs treated with 33 nM Dofetilide (middle), and ACMs treated with 100 nM Dofetilide (bottom). **(E)** Histogram representing the proportion of arrhythmic cells (AI > 20) in control ACMs and ACMs treated with 33 nM and 100 nM Dofetilide. **(F)** Quantification of KS-D between control or Dofetilide-treated ACMs conditions. P-value *** < 0.0001.

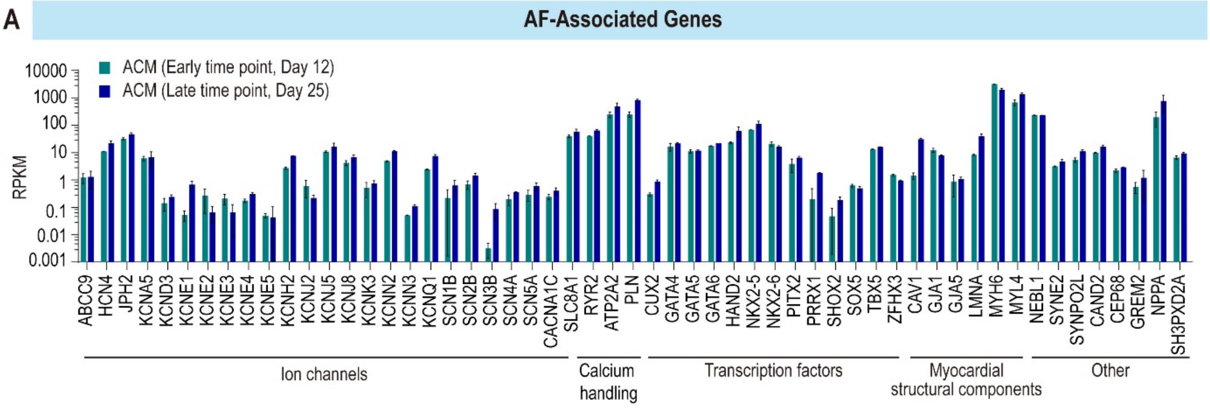

B AF-Association Gene Candidate Table

Rare variant	GWAS Locus		
		**Ion Channels**	
×	×	*HCN4*	*Pacemaker current; I_f*
×		*KCNA5*	*Voltage-gated K^+ channel; Kv1.5; I_{Kur}*
×	×	*KCND3*	*Voltage-gated K^+ channel; Kv4.3; I_{to}*
×	×	*KCNJ5*	*Inwardly rectifying K^+; Kir3.4; I_{KACh}*
×	×	*KCNN3*	*Small conductance, Ca^{2+}-activated K^+ channel; KCa2.2; I_{KCa}*
		**Transcription Factors**	
×	×	*GATA4*	*GATA-binding protein 4*
×	×	*GATA5*	*GATA-binding protein 5*
×	×	*GATA6*	*GATA-binding protein 6*
×	×	*HAND2*	*Heart- and neural crest derivatives-expressed protein 2*
×	×	*NKX2-5*	*Homeobox protein NKX2-5*
×	×	*NKX2-6*	*Homeobox protein NKX2-6*
×	×	*PITX2*	*Paired-like homeodomain protein 2*
×	×	*TBX5*	*T-box protein 5*
×	×	*ZFHX3*	*Zinc finger homeobox protein 3*
		**Myocardial Structural Components**	
×	×	*GJA1*	*Gap junction protein, connexin43*
×	×	*GJA5*	*Gap junction protein, connexin40*
×	×	*SYNE2*	*Nuclear envelope protein, spectrin repeat containing nuclear envelope protein 2*
×	×	*PLN*	*Phosphlamban, sarcoplasmic reticulum Ca^{2+}-ATPase*
		**Signaling**	
×		*NPPA*	*Natriuretic peptide precursor A*
×	×	*SH3PXD2A*	*Tyrosine kinase substrate, SH3 and PX domains 2*

C Effect of AFib-associated genes on APD

Gene	Normalized APD75	Normalized Systolic Interval
siGATA5	1.22	1.09
siGATA6	1.18	0.98
siPITX2	1.17	1.09
siKCNA5	1.16	1.4
siGATA4	1.14	1.12
siKCNJ5	1.14	1.26
siHCN4	1.11	1.08
siGJA1	1.07	1.18
siTBX5	1.06	1.28
siSYNE2	1.06	0.82
siNKX2-6	1.04	1.16
siSH3PXD2A	1.02	0.99
siKCNN3	0.99	1.3
siNPPA	0.98	
siZFHX3	0.98	1.14
siNKX2-5	0.97	
siHAND2	0.96	1.33
siGJA5	0.95	1.18
siKCND3	0.9	0.82
siPLN	0.87	0.77

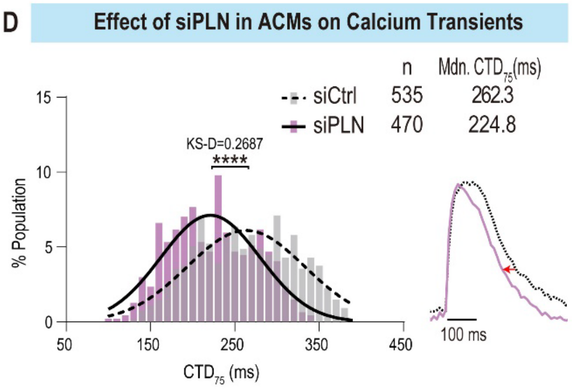

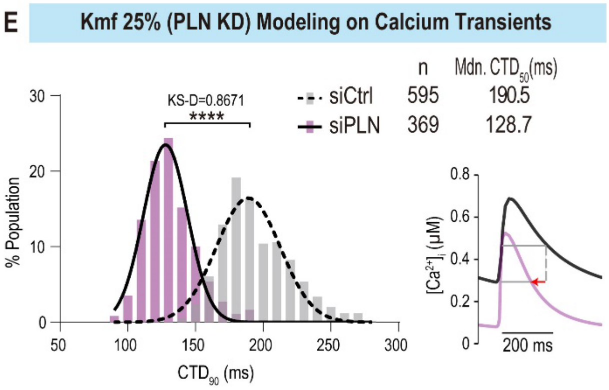

Fig. S3. Selection of AF-associated genes for phenotypical characterization using our novel HT platform. (A) Histogram representing expression level (RPKM) of previously known AF-associated genes using RNAseq data from Day 12 and Day 25 ACMs. **(B)** Selection of 20 AF-genes harboring rare variant in familial AF studies and/or SNPs reported in GWAS studies. **(C)** Numerical values of data presented in heat map

Disease Models & Mechanisms • Supplementary information

in Fig.3A. APD75 and systolic Intervals were normalized to their respective controls. In flies there is no homolog for NPPA and tinman/Nkx2.5 KD flies fail to develop hearts. **(D)** Histogram showing the distribution of calcium transient duration (CTD) values in siControl and siPLN condition in ACMs (right) and representative single calcium transients for both conditions (left). **(E)** Histogram of CTD generated from HAMs (left) and representative traces (right) in response to Kmf25% condition (=PLN KD). P-value *** < 0.0001.

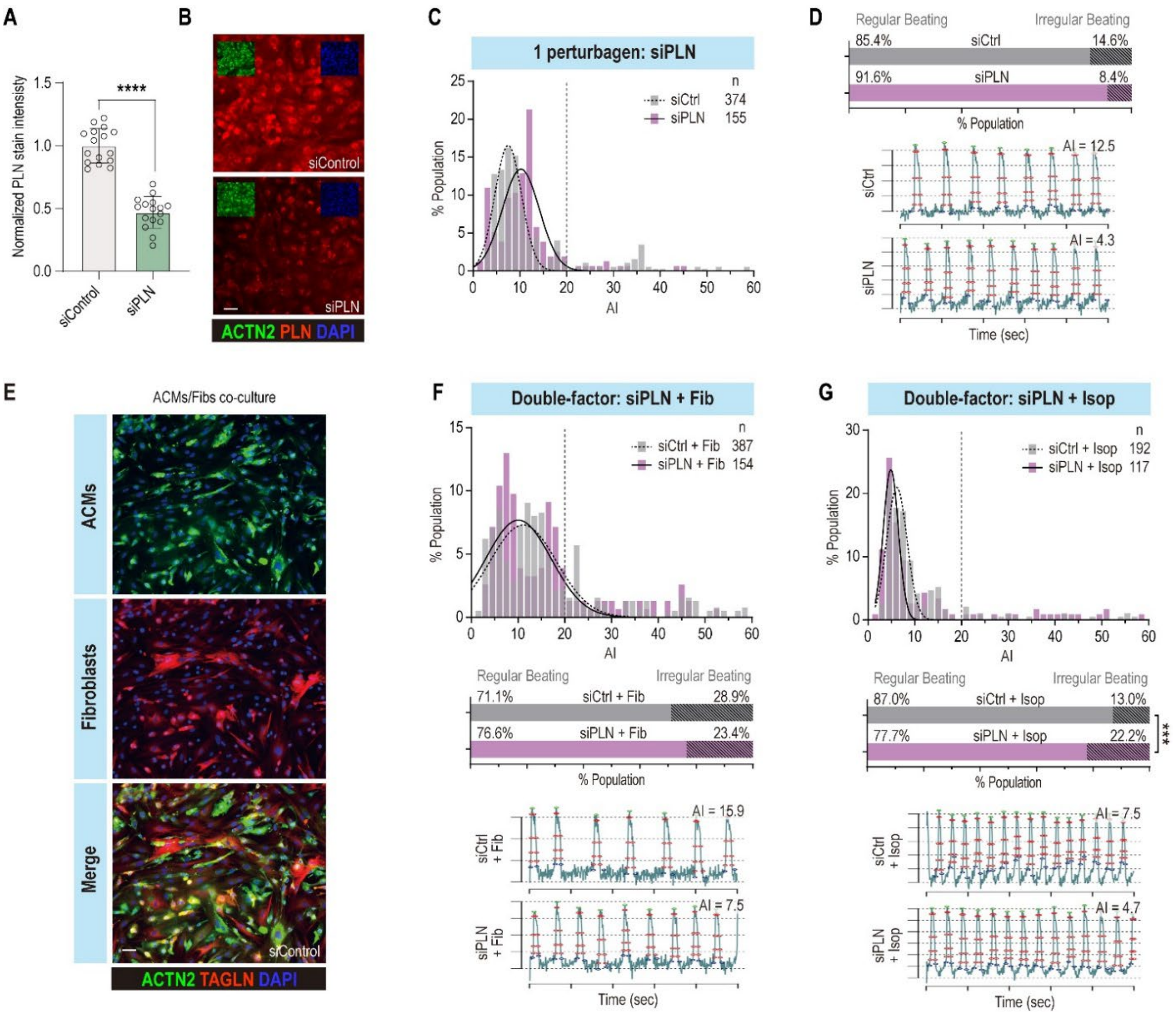

Fig. S4. Testing of multiple perturbagens to trigger arrhythmia in ACMs. (A) Histogram showing the quantification of PLN staining intensity in siControl and siPLN conditions in ACMs. **(B)** Representative images of ACMs stained for actinin2 (ACTN2, green) to mark ACMs and for phospholamban (PLN, red) showing a significant reduction of PLN protein levels upon PLN KD. **(C)** Histogram showing the distribution of AI values from ACMs in siControl and siPLN conditions. **(D)** Quantification of irregular AP peak train (top). Representative AP traces in siControl and siPLN conditions. **(E)** Representative images of ACMs co-cultured with human dermal fibroblasts and stained with ACTN2 (cardiac, green) and TAGLN (fibroblasts, red). **(F)** Histogram showing the distribution of AI values from ACMs co-cultured with fibroblasts (Fib) in siControl and siPLN conditions (top). (Middle) Quantification of the percentage of ACMs ttwith irregular AP peak trains for each condition. (Bottom) Representative AP peak trains for each condition. (G) Histogram showing the distribution of AI values from ACMs treated with Isop in siControl and siPLN conditions (top). (Middle) Quantification of the percentage of ACMs with irregular AP peak trains for each condition. (Bottom) Representative AP peak trains for each condition.

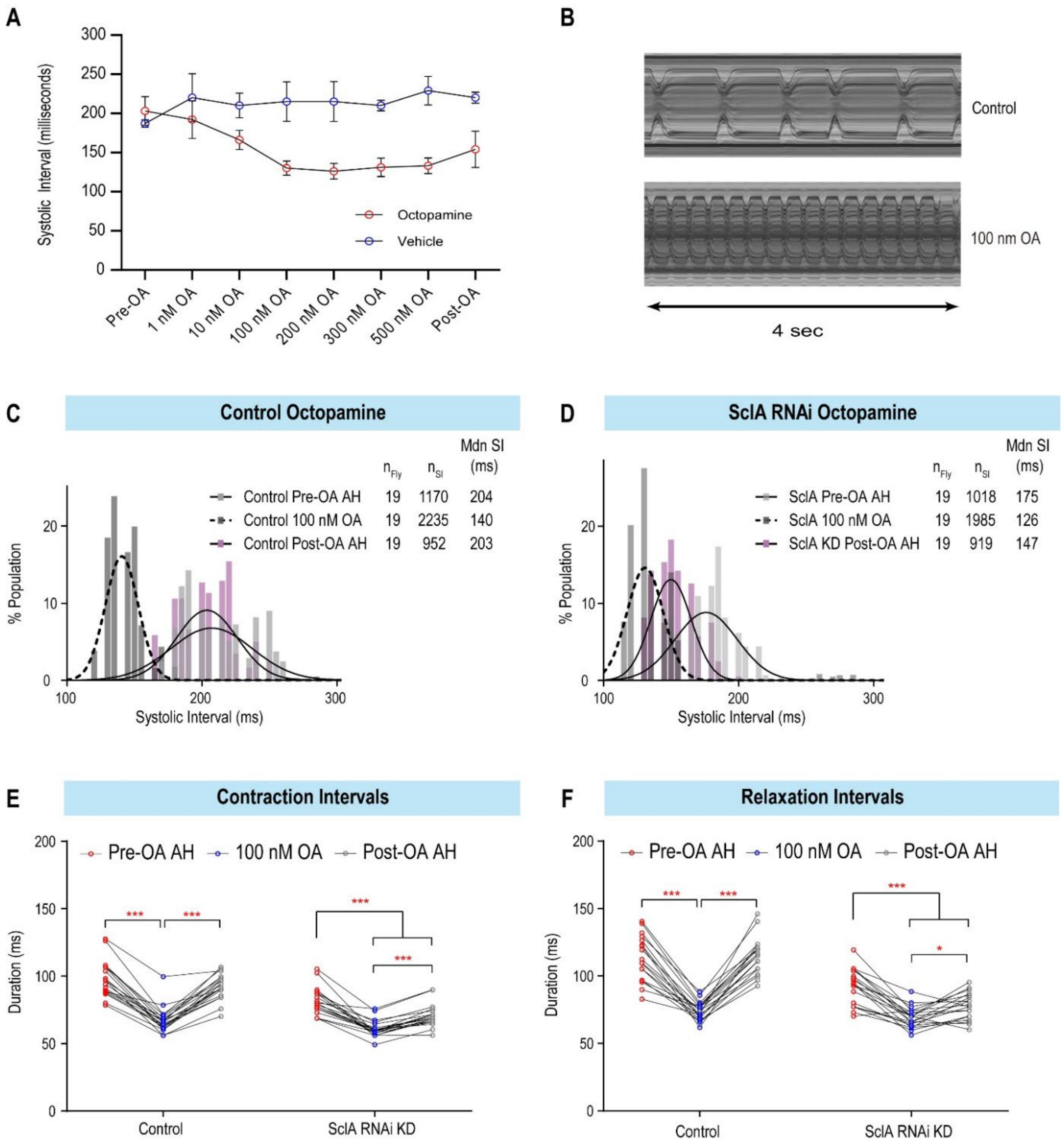

Fig. S5. Optimization of octopamine treatment in flies (A) Graph showing average SI values in response to escalating doses of OA in flies. **(B)** Representative M-modes from one heart before (top) and during application of 100nM OA (bottom) showing dramatic increases in heart rate. **(C)** Histograms showing the distribution of SI values in control hearts pre-OA pacing, during exposure to 100nM OA, and 15 min post-OA application. Distribution of SIs post-OA returns to that of pre-OA. **(D)** showing the distribution of SI values in SclA KD hearts pre-OA pacing, during exposure to 100nM OA, and 15 min post-OA application. Distribution of SIs post-OA are significantly shorter compared to pre-OA and are due to decreases in both **(E)** the contraction and **(F)** relaxation phases of the systolic intervals(p-value < 0.05, repeated measures 2-way ANOVA).

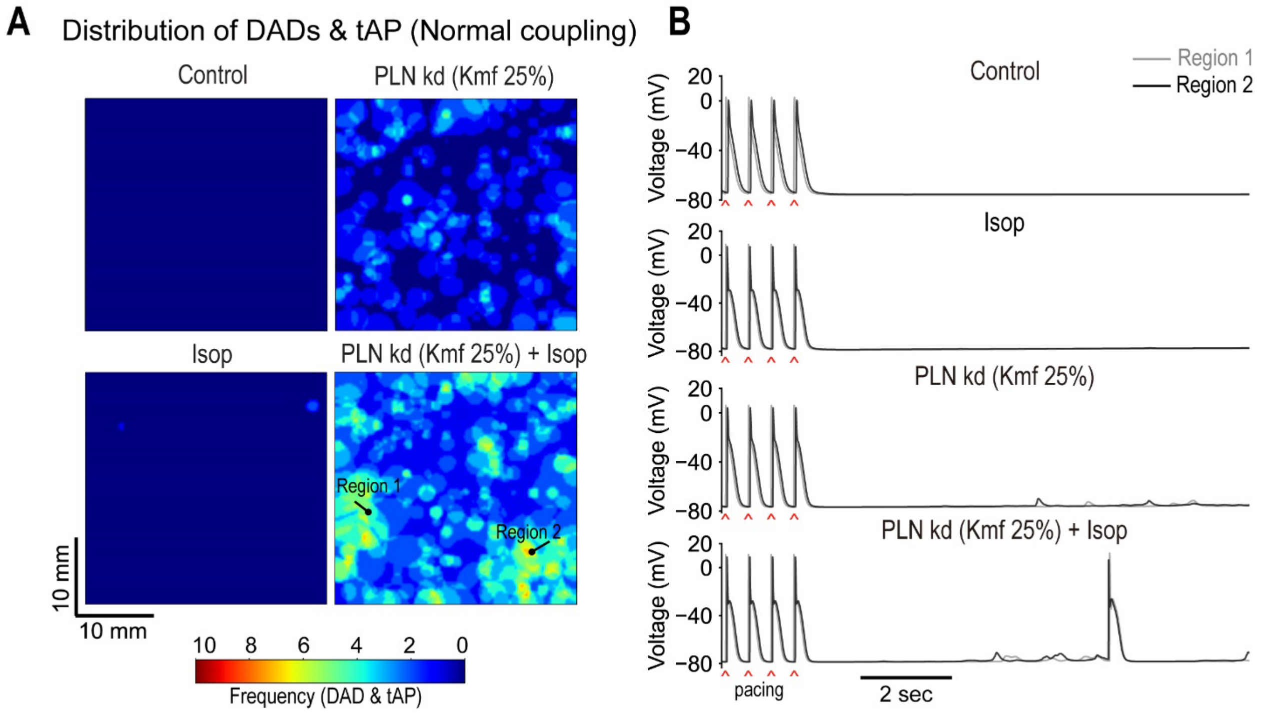

Fig. S6. Effects of PLN kd (Kmf 25%) and Isop on the triggered activity in heterogeneous human atrial tissue with normal cell-to-cell coupling. Tissue was paced at 2 Hz for 10 s followed by a period of 10 s without stimulation. **(A)** Spatial distribution of DADs and tAPs in the atrial tissue for PLN KD (Kmf 25%) and after Isop treatment. **(B)** Superimposed traces of APs from two regions (marked in panel ***A***) of the atrial tissue with normal cell-to-cell coupling for each perturbation.

Disease Models & Mechanisms • Supplementary information

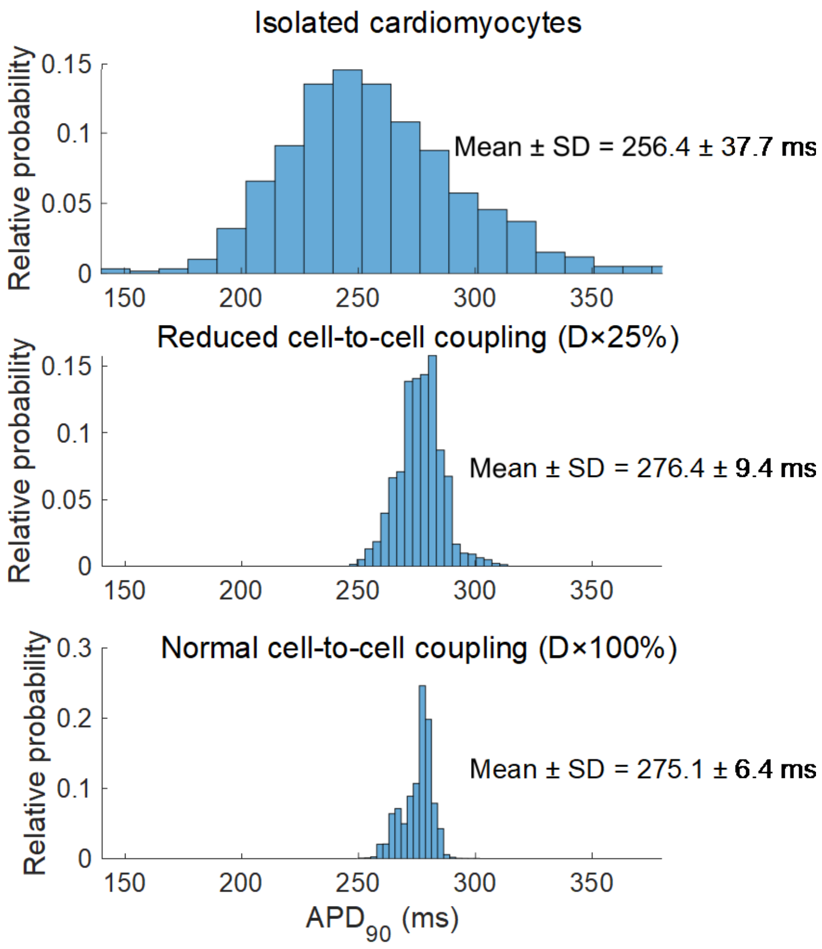

Fig. S7. Normalized histogram plot illustrating distribution of APD90 for the human atrial population of (top) isolated cardiomyocytes and coupled cardiomyocytes in tissue with (middle) reduced, or (bottom) normal cell-to-cell coupling. The mean and SD values are indicated in each panel.

Disease Models & Mechanisms • Supplementary information

Table S1. Human AF candidate genes tested in ACMs (Fig. S2) are shown with their *Drosophila* orthologs. Studies demonstrating the presence and/or function of these orthologs in the fly heart are listed and genes common to both ACMs and fly hearts are bolded. Data from tissue-specific RNA Seq analysis and curated in FlyAtlas2 (https://flyatlas.gla.ac.uk/FlyAtlas2/) shows cardiac expression for approximately half of the genes in the table (indicated by +). Note that many of the ion channels and transcription factors (TF) that have been shown to be functional in the heart did not show up in the Fly Atlas dataset and none showed up in the cardiac proteomic analysis by Cammarato et al (2011, PMID: 21541028), likely because expression in the heart is too low relative to other structural genes (e.g. Myosin heavy chain, Mhc).

CATEGORY	GENE	FLY ORTHOLOG	Reference	FlyAtlas2
CHANNEL	**ABCC9**	**dSur**	Akasaka, et al 2006 -PMID: 16882722; Eleftherianos et al, 2011 - PMID: 21719711	-
CHANNEL	**HCN4**	**Ih**	Monier, et al, 2005 - PMID 16284119	+
CHANNEL	**JPH2**	**junctophilin**		+
CHANNEL	**KCNA5**	**Shaker**	Ocorr et al, 2017 - PMID: 28542428	-
CHANNEL	**KCND3**	**Shal**	Ocorr et al, 2017 - PMID: 28542428	-
CHANNEL	KCNE1 - 5	No ortholog	KCNQ works without MinK	
CHANNEL	**KCNH2**	**seizure**	Ocorr et al, 2017 - PMID:28542428	-
CHANNEL	**KCNJ2, 5, 8**	**Irk**	Ocorr et al, 2017 - PMID:28542428	-
CHANNEL	**KCNK3**	**ork / sandman**	LaLevee et al – PMID: 16890525 Klassen et al - PMID: 28328397	- / -
CHANNEL	**KCNN2, 3**	**SK**		+
CHANNEL	**KCNMA**	**BK – not tested in ACMs?**	Pineda et al, - PMID: 33629867	+
CHANNEL	**KCNQ1**	**KCNQ**	Ocorr et al, 2007 - PMID: 17360457	-
CHANNEL	**RYR2**	**RyR**	Lin et al, PMID: 21493892	+
CHANNEL	**SCN1 - 5**	**nap**	Dowse et al, PMID: 8719771 Ganetsky, PMID: 2420953	+
TF	**CUX2**	**cut**	Blochlinger et al, PMID 8330519 Zappia et al, PMID 32815271	-
TF	**GATA4 / 5 / 6**	**pnr / grn / GATAd**	Klinedinst & Bodmer, 2003 - PMID: 12756184	+ / + / +
TF	**HAND2**	**Hand**	Kolsch and Paululat, 2002 - PMID: 12424518 Jonhson et al 2011 - PMID: 21965617	+
TF	**NKX2-5 / 2-6**	**tin**	Bodmer et al 1990 - PMID: 7915669	-
TF	PITX2	Ptx1		-
TF	PRRX1	CG9876		-
TF	SHOX2	CG34367		-
TF	**SOX5**	**Sox102F**		+
TF	**TBX5**	**Bifid / Doc1 / Doc2 / Doc3**	Bi - Ahmad et al 2012 - PMID: 22814603 DOC -Reim et al 2003 - PMID: 12783790 Berkeley Drosophila Genome Project	- / - /- /-
TF	ZFHX3	zfh2		-
MYOCARDIAL	CAV1	No ortholog		
MYOCARDIAL	**GJA1**	**CG11459 / 26-29-p / CG4847**	Cammarato et al 2011 - PMID: 21541028	+
MYOCARDIAL	GJA5	No ortholog		
MYOCARDIAL	**LMNA**	**LamC / Lam**	Cammarato et al 2011 - PMID: 21541028	+

Disease Models & Mechanisms • Supplementary information

MYOCARDIAL	**MYH6**	**Mhc**	Lovato et al, 2002 - PMID: 12397110 Cammarato et al, 2011 - PMID: 21541028	+
MYOCARDIAL	**MYL4**	**Mlc-c / Mlc1**	Cammarato et al 2011 - PMID: 21541028	+
MYOCARDIAL	**NEBL**	**Lasp**	Cammarato et al 2011 - PMID: 21541028	+
MYOCARDIAL	**SYNE2**	**Msp300**	Cammarato et al 2011 - PMID: 21541028	+
MYOCARDIAL	**SYNPO2L**	**CG1674**	Cammarato et al 2011 - PMID: 21541028	+
OTHER	**C9ORF3**	**CG10602**		+
OTHER	**CAND2**	**Cand1**		+
OTHER	CEP68	No ortholog		
OTHER	GREM2	No ortholog		
OTHER	**NEURL**	**neur**		+
OTHER	NPPA	No ortholog		
OTHER	**SH3PXD2A**	**cindr / Nipped-A**	Cammarato et al 2011 - PMID: 21541028	+ / +

Disease Models & Mechanisms • Supplementary information

Table S2. Screen results in ACMs. Table displays Median, Normalized Median, KS-D and P- values related to Fig.3A.

Gene ID	Mdn. APD75	Norm Median APD75	KSD	P-value of KS-D
Ctrl	133.6	1		
GATA4	151.8	1.136227545	0.1577	<0.0001
GATA5	162.6	1.217065868	0.2691	<0.0001
GATA6	157.8	1.181137725	0.2482	<0.0001
GJA1	142.5	1.066616766	0.05343	0.0019
GJA5	127.4	0.953592814	0.2149	<0.0001
HAND2	127.6	0.95508982	0.2466	<0.0001
HCN4	148.4	1.110778443	0.1756	<0.0001
KCNA5	155.5	1.163922156	0.2014	<0.0001
KCND3	119.6	0.895209581	0.2371	<0.0001
KCNJ5	152.4	1.140718563	0.1893	<0.0001
KCNN3	132.4	0.991017964	0.1503	<0.0001
NKX2-5	129.9	0.972305389	0.1762	<0.0001
NKX2-6	138.3	1.035179641	0.04106	0.0275
NPPA	131.4	0.983532934	0.1047	<0.0001
PITX2	156	1.167664671	0.2053	<0.0001
PLN	116.1	0.869011976	0.3305	<0.0001

Table S3. Screen results in flies. Table displays Normalized systolic interval (SI), standard deviations and P values related to Fig.3A.

fly_gene	Norm SI	Deviations	P-Value
Sh	1.40333138	0.40333138	6.91^{E}-10
Hand	1.32601473	0.32601473	2.52^{E}-09
SclA	0.77328487	0.22671513	4.93^{E}-09
Sk	1.25795515	0.25795515	4.99^{E}-08
Doc1	1.26981919	0.26981919	4.57^{E}-07
Pnr	1.12001245	0.12001245	5.21^{E}-06
Shal	0.81835564	0.18164436	1.31^{E}-05
Irk3	1.30245197	0.30245197	1.55^{E}-05
shakb	1.15642458	0.15642458	1.18^{E}-04
Scro	1.16119611	0.16119611	0.00021027
MSP300	0.81943812	0.18056188	0.00046931
Ih	1.07869321	0.07869321	0.01360059
Ptx1	1.08533093	0.08533093	0.0543814
Zfh-2	1.14018008	0.14018008	0.08787157
Cindr	0.9867872	0.0132128	0.71808748

Disease Models & Mechanisms • Supplementary information

Table S4. Glossary for model parameters that were perturbed for constructing populations of human atrial models.

Parameter	Note
G_{Na}	Fast Na^+ current, maximal conductance
G_{CaL}	L-type Ca^{2+}current, maximal conductance
G_{to}	Transient outward K^+ current, maximal conductance
G_{Kur}	Ultra-rapid delayed rectifier K^+ current, maximal conductance
G_{Kr}	Rapid delayed rectifier K^+ current, maximal conductance
G_{Ks}	Slow delayed rectifier K^+ current, maximal conductance
G_{K1}	Inward rectifier K^+ current, maximal conductance
G_{Kp}	Conductance of the plateau K^+ current
G_{NaB}	Background Na^+ current, maximal conductance
G_{CaB}	Background Ca^{2+} current, maximal conductance
G_{CaP}	Sarcoplasmic Ca^{2+} pump current, maximal pump rate
G_{ClCa}	Ca^{2+} activated Cl^- current, maximal conductance
G_{ClB}	Background Cl^- current, maximal conductance
V_{NCX}	Na^+/Ca^{2+} exchange current, maximal exchange rate
V_{NaK}	Na^+/K^+ pump current, maximal pump rate
V_{SERCA}	Rate of the SERCA pump
$V_{RyR,Rel}$	Rate of the SR Ca^{2+} release via ryanodine receptors
$V_{RyR,Leak}$	Rate of the SR Ca^{2+} leak via ryanodine receptors

Table S5. Conduction velocity measured in the 2D tissue.

	Conduction velocity	
Pacing rate	1 Hz	2 Hz
Normal coupling (D×100%)	0.63 m/s	0.41 m/s
Reduced coupling (D×25%)	0.28 m/s	0.19 m/s

Disease Models & Mechanisms • Supplementary information

Table S6. Human AF candidate genes tested in the fly heart (Fig. S2) are listed with their human orthologs and the stock center ID number.

Fly RNAi	Human Ortholog	Stock #	Stock #2	Source
**Bifid**	TBX 2,3	100598	330228	VDRC
**Cindr**	SH3KBP1	38854	330411	VDRC
**Doc1**	TBX6	16746	104927	VDRC
**Doc2**	TBX6	103431		VDRC
**Doc3**	TBX6	30550	104922	VDRC
**GATAd**	GATA1	100389		VDRC
**GATAe**	GATA4	10418		VDRC
**Grain**	GATA2,3	105192	330376	VDRC
**Hand**	Hand	23306	330058	VDRC
**Ih**	HCN2-4	110274	29574	VDRC
**Irk1**	KCNJ2,4,12,18	107389	28431	VDRC
**Irk2**	KCNJ2,4,12,18	4341	108140	VDRC
**Irk3**	KCNJ10,15	3886	101174	VDRC
**MSP300**	SYNE1	107183	25906	VDRC
**Pnr**	GATA4,5,6	6224	101522	VDRC
**Pnr**	GATA4,5,6	34659	33744	BDSC
**Ptx1**	Ptx1-3	19831	107785	VDRC
**SclA**	PLN	28957		BDSC
**SclA/B**	PLN	62935	28957	BDSC
**Scro**	NKX2.1,2.4	33902	330398	VDRC
**Sh**	KCNA1-5	23673	104474	VDRC
**Shal**	KCND1-3	103363	330383	VDRC
**Sk**	KCNN1-3	2855	7052	VDRC
**Sk**	KCNN1-3	27238	53881	BDSC
**Tin**	NKX2.5	190512	101825	VDRC
**Twist**	TWIST1, 2	37091	37092	VDRC
**Zfh-2**	ZFH2-4	13305	110784	VDRC

Disease Models & Mechanisms • Supplementary information

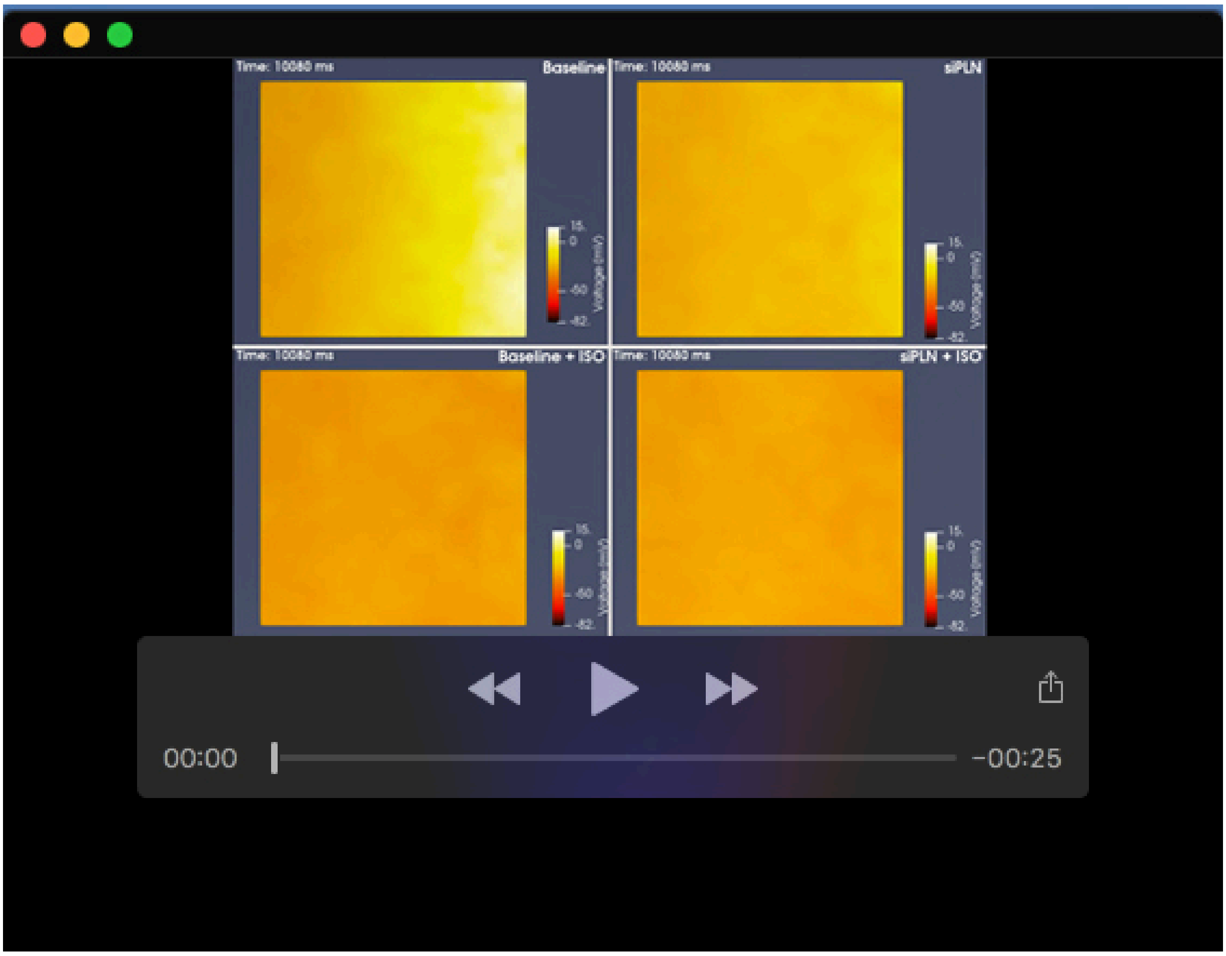

Movie 1. Simulated tissue (normal tissue coupling) voltage map showing AP wave propagation elicited by the last pacing stimulus (t=10,000 ms) and the membrane voltage activity thereafter. Simulated conditions are: top left – baseline; bottom left – baseline + ISO; top right – PLN knockdown; bottom right – PLN knockdown + ISO.

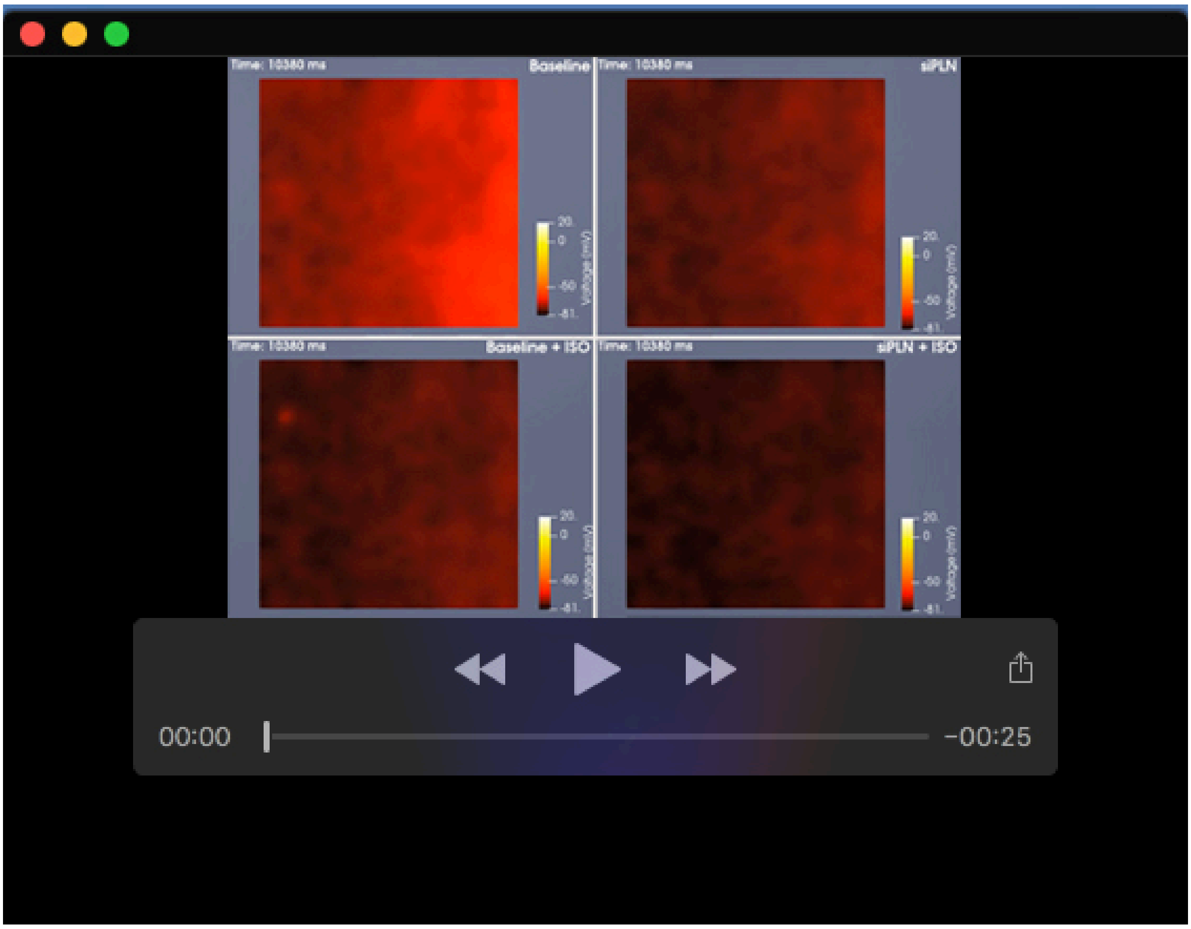

Movie 2. Simulated tissue (reduced tissue coupling D×25%) voltage map showing AP wave propagation elicited by the last pacing stimulus (t=10,000 ms) and the membrane voltage activity thereafter. Simulated conditions are: top left – baseline; bottom left – baseline + ISO; top right – PLN knockdown; bottom right – PLN knockdown + ISO.
